# Feather Microbiota Landscapes: Biogeography and Phenology Shape Feather Microbiota Traits in a Migratory Seabird in a Subantarctic Ecosystem

**DOI:** 10.1111/mec.70114

**Published:** 2025-10-03

**Authors:** Manuel Ochoa‐Sánchez, Eliana Paola Acuña‐Gómez, Claudio A. Moraga, Jorge Acevedo, Pedro Valenzuela, Luis E. Eguiarte, Valeria Souza

**Affiliations:** ^1^ Departamento de Ecología Evolutiva Instituto de Ecología, Universidad Nacional Autónoma de México Ciudad de México México; ^2^ Centro de Estudios del Cuaternario de Fuego, Patagonia y Antártica (CEQUA) Punta Arenas Chile; ^3^ Posgrado en Ciencias Biológicas, Unidad de Posgrado Universidad Nacional Autónoma de México Ciudad de México México

**Keywords:** avian microbiota, environmental heterogeneity, microbial biogeography, microbial dispersal, seasonal microbiota, theory of island biogeography, wildlife microbiota

## Abstract

It is known that phenological changes (i.e., behavioural and sometimes morphological and physiological traits that repeat annually) influence the wildlife gut microbiota. However, it remains largely unknown to what extent geographic variation could modulate the effect that phenology has on wildlife microbiota. Here, we analysed the feather microbiota in adult Magellanic penguins ( *Spheniscus magellanicus* ) and the microbes from samples of nest soil and seawater, using Illumina MiSeq sequencing of bacterial 16S rRNA (V3–V4 variable region) at three phenological stages (courtship, egg‐laying and chick‐rearing) across five nesting colonies with environmental heterogeneity, in the Magellan Strait, Chile. We found over 67,000 ASVs, most belonging to the bacterial family *Moraxellaceae*. We detected seven core bacterial genera despite geographic and phenological variation; among them, *Psychrobacter* had the highest relative abundance. Phenology affected feather microbiota alpha diversity and the relative abundance of selected genera in a colony‐specific fashion. Still, it consistently affected feather and nest soil microbial composition, highlighting a phenological microbial succession pattern in penguin feathers and nest soil. From the geographic perspective, we detected three main results in the penguin feather microbiota: (1) alpha diversity was higher in the largest colonies, although only in the chick‐rearing stage; (2) a significant distance‐decay pattern, in the egg‐laying and chick‐rearing stages; and (3) compositional clusters that follow the geographic location of each colony. Our results highlight how temporal and environmental heterogeneity shape microbial traits in marine wildlife.

## 1 Introduction

Seasonality refers to the periodic variations that the environment experiences annually. From the biological perspective, wildlife undergoes behavioural and sometimes morphological, physiological, and colour changes that repeat annually, commonly referred to as phenology (Chuine and Regniere 2017). In the tropics, seasonal temperature changes are subtle, whereas significant changes in precipitation occur across months, sites, and interannually (Feng et al. 2013). In contrast, seasonality comprises major abiotic changes across months in subantarctic environments. For example, in Southern Patagonia, there are strong seasonal changes in temperature across the region (Aguirre et al. 2018; Weidemann et al. 2018). For instance, in Punta Arenas (the biggest city in the southernmost region of South America), temperatures go from > 10°C during the austral summer to 2°C during the austral winter, whereas precipitation and wind speed follow a dynamic pattern across the Magellan region (Aguirre et al. 2018; Weidemann et al. 2018).

Seasonality has a major influence on the composition and diversity of the environmental microbial pool. At a global scale, seasonal temperature and precipitation changes drive the composition and diversity of soil and marine microbiota (Ward et al. 2017; Zhao et al. 2022). In subantarctic environments, seasonality influences the relative abundance of certain microbial taxa (Varliero et al. 2024). Also, in Antarctic and subantarctic ecosystems, it is expected that the relative abundance of psychrophilic taxa (i.e., organisms that thrive under cold conditions) with narrow thermic niches should undergo seasonal decreases, while the relative abundance of taxa with wide thermic niches should increase (Margesin and Miteva 2011). However, the effect of seasonality on soil microbiota might be buffered by the vegetation cover, since the vegetation cover could decrease surface temperature by energy dissipation (Feldman et al. 2023).

The effect of seasonality on environmental microbial pools is shaped by changes in abiotic factors and the particular environmental characteristics of each ecosystem. In wild host‐associated microbiota, phenological stages are associated with changes in the gut microbiota (Baniel et al. 2021; Tang et al. 2023). Interestingly, behavioural changes associated with environmental seasonality can produce modifications in the skin microbiota of amphibians (Tong et al. 2020) and mammals (Van Cise et al. 2020; Li, Li, et al. 2023).

Recent advances in microbiology have moved us further from the popular paradigm ‘everything is everywhere, but the environment selects’ (Baas‐Becking 1934) to understand how abiotic and biotic factors geographically structure the composition of microbial communities (Martiny et al. 2006). For instance, differences in microbial composition in the gut microbiota of birds have been detected both at microgeographic and continental scales (Klomp et al. 2008; van Veelen et al. 2023). Likely, host‐associated microbiota assembly is a process influenced simultaneously by geographic structure and microbial dispersal, given that the host experiences environmental variability along its distribution, and there are appropriate ways for microbes to disperse. For example, site‐specific environmental microbial recruitment could produce structure in host‐associated microbiotas along their distribution/migration. This has been shown in the surface and gut microbiota of some bird species (Ochoa‐Sánchez, Acuña‐Gómez, et al. 2024; Risely et al. 2018; Grond et al. 2023). However, host‐associated microbiota assembly could also be homogenised, given that the host experiences abiotic conditions that facilitate microbial dispersal, such as strong winds (Chen et al. 2021).

The Magellanic penguin ( *Spheniscus magellanicus* ) is a migratory seabird with a wide latitudinal distribution in southern South America (Boersma et al. 2013). It has a seasonal breeding phenological cycle that starts with courtship (October), continues with the egg‐laying stage (December) and finishes with the chick‐rearing stage (January–February) (Boersma et al. 2013). During their breeding phenology, Magellanic penguins experience variation in environmental exposure. During courtship and chick‐rearing, penguins have the greatest environmental exposure since they forage constantly to obtain enough resources to lay the egg and feed the chick, respectively. However, during the egg‐laying stage, penguins experience much less environmental exposure since they are incubating their eggs. Unlike other penguin species (e.g., king and emperor penguins), Magellanic penguins alternate incubation; hence, at some point, both species could be inside the nest before doing the ‘incubation shift’ when parents alternate. Also, Magellanic penguins use their burrows to rest, so it is common to find both parents inside the nest with their egg. This breeding cycle occurs within the austral environmental seasonality; hence, as the Magellanic penguin phenology progresses, there are changes in temperature, relative humidity, and wind speed in their colonies.

The Magellanic penguin is an ideal species to test the effect of geography since it inhabits colonies with contrasting vegetation cover and different climate regimes (Ochoa‐Sánchez, Acevedo, et al. 2024). Furthermore, the Magellanic penguin nesting colonies have an insular distribution, representing an interesting example to test classic island biogeographic predictions: (1) Larger islands have more species and (2) Islands farther away from the continent have fewer species (MacArthur and Wilson 1967). A positive relationship between area and microbial diversity has been detected in several island‐like systems, such as the hole area of Antarctic cryoconite holes (Darcy et al. 2018); forest fragment size (Vannette et al. 2016) and host plant size (Dinnage et al. 2019), yet to the best of our knowledge, this hypothesis has not been tested in animal hosts. Here, we use microbial alpha diversity and compositional similarity to address the following predictions: (1) Larger islands harbour penguins with higher feather microbial alpha diversity but less feather microbial similarity in comparison with penguins from smaller islands; and (2) Islands farther away from the continent harbour penguins with less feather microbial compositional similarity (i.e., feather microbiota experiences higher compositional heterogeneity).

In this study, we investigated the effect of phenology and biogeography on the feather and associated environmental microbiota of Magellanic penguins in five nesting colonies in the Magellan Strait, Chile, through three phenological breeding stages: courtship, egg‐laying, and chick‐rearing. We analysed the bacterial communities of the penguin plumage and environmental samples using high‐throughput sequencing of the hypervariable region V3–V4 of the 16S rRNA gene. Our research hypothesis was the following: given the seasonal abiotic changes and behavioural variation that occur throughout the Magellanic penguin breeding phenology, we expect that both alpha diversity and microbial composition of the feather and nest soil microbiota would vary across phenological stages. Alternatively, if phenological variation is not related to the feather microbiota, then feather alpha diversity and microbial composition should be constant across phenological stages.

## 2 Materials and Methods

### 2.1 Study Area

**FIGURE 1:**
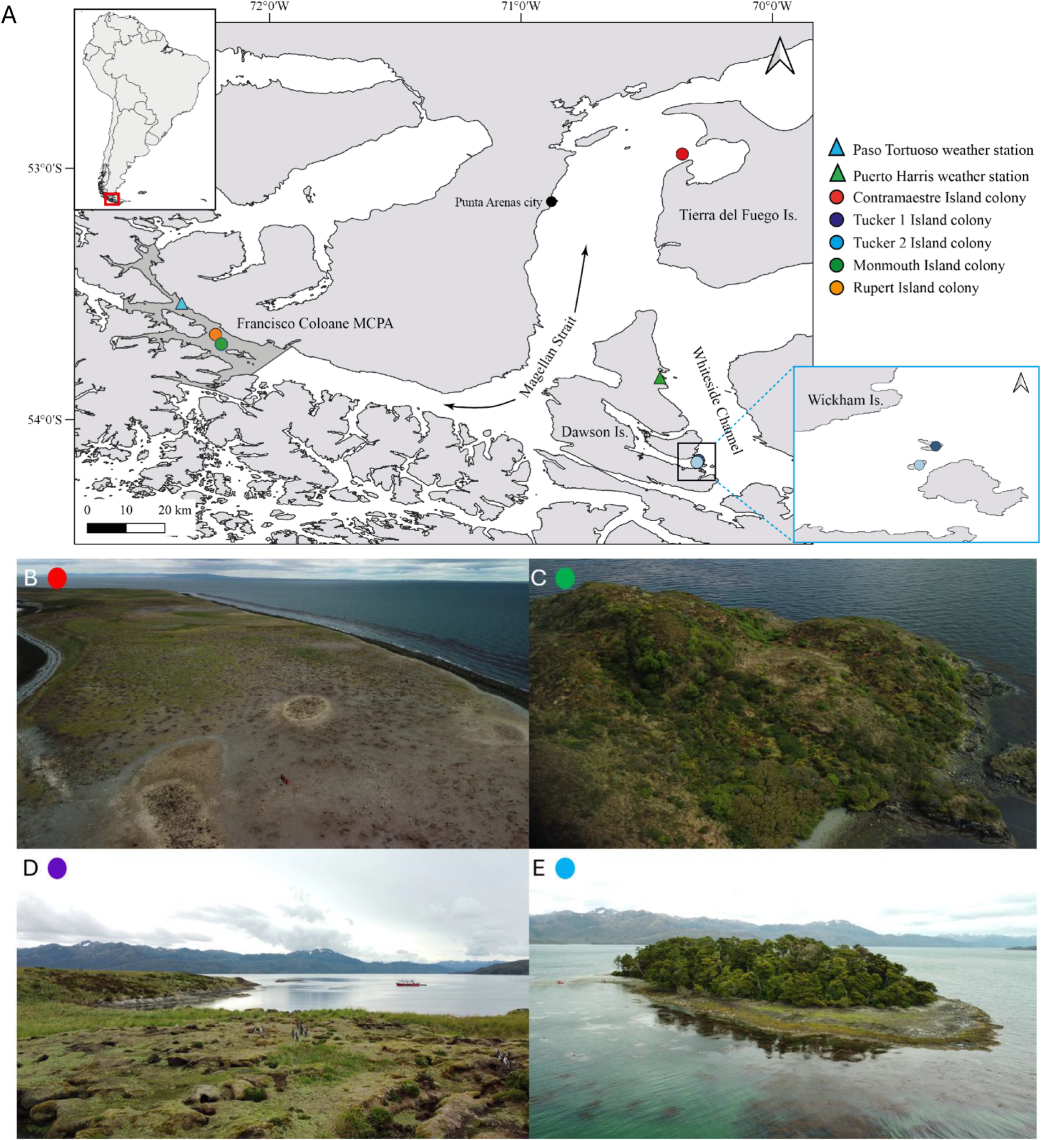
Geographic distribution and general characteristics of subantarctic Magellanic penguin breeding colonies. (A) Map showing the location of Magellanic penguin breeding colonies (circles) across the Magellan Strait. The photos below show general features of Magellanic penguin breeding colonies: (B) Contramaestre, (C) Monmouth, (D) Tuckers 1 and (E) Tuckers 2. Triangles indicate the location of the weather stations. Note that in the Contramaestre colony, we also had access to a weather station, but we did not add a weather station symbol for the sake of clarity. We did not add a photo of the Rupert colony, since its general features are similar to the Monmouth colony. Photo credits belong to Pedro Valenzuela.

The sampled nesting colonies are characterised by different climatic regimes and contrasting environmental characteristics (refer to Table 1, Figure 1). The Contramaestre Island colony is located in the eastern portion of the Magellan Strait towards the Atlantic mouth. Its climate regime is cold steppe, and it lacks a dense vegetation cover. The Tuckers islets are two breeding colonies located southwards of Dawson Island, in the Whiteside Channel, Tierra del Fuego, Chile. The Tuckers islets are neighbour colonies separated by ca. 500 m and are characterised by a climate regime of tundra, yet each colony has a different vegetation cover. Interestingly, Magellanic penguins inhabiting the Tuckers 2 colony have their burrows under living trees and even inside fallen trees, an atypical nest site for this species (Ochoa‐Sánchez, Acuña‐Gómez, et al. 2024). Finally, the Monmouth and Rupert colonies are located towards the Pacific mouth of the Strait. These colonies are within the Marine Protected Area ‘Francisco Coloane’ and are subject to a cold‐temperate climate regime.

**TABLE 1:** Biological features and environmental characteristics of subantarctic Magellanic penguin breeding colonies sampled in this study.

Colony	Island area (ha)	Population size (reproductive pairs)	Sampled phenological stages	Vegetation cover
Contramaestre	87.5	ca. 13,000 (CEQUA 2018)	Courtship	Heterogenous vegetation. Patches of creeping vegetation, coastal grassland and shrubs (*Chiliotrichum diffusum* and *Festuca gracilima*)
			Chick‐rearing	
Monmouth	45.8	316 (Acevedo et al. 2007) 42 (Acevedo et al. 2014)	Egg‐laying	Heterogeneous vegetation. Patches of tall grasses (family: Poaceae) and shrubs (*Gaultheria mucronata* and *Hebe elliptica*) alternating with *Nothofagus betuloides* and *Drimys winteri* forest
			Chick‐rearing	
Rupert	72	22,500 (August 2005)/6,994 (January–April 2007) (Miranda et al. 2009)	Courtship	Heterogeneous vegetation. Patches of grasses (family: Poaceae), shrubs (*G. mucronata* and *H. elliptica* ) and *Sphagnum* spp. mosses
			Egg‐laying	
			Chick‐rearing	
Tuckers 1	9.55	2,218 (Acevedo et al. 2024)	Courtship	Heterogeneous vegetation cover. Patches of tall grasses (family: Poaceae), shrubs (Figure 2A) and the remains of desiccated dead trees (likely the remains of *Nothofagus* spp.).
			Egg‐laying	
			Chick‐rearing	
Tuckers 2	7.2	No available data	Courtship	Dense forest of *Nothofagus* spp.
			Egg‐laying	
			Chick‐rearing	

### 2.2 Environmental Data Collection

To characterise environmental conditions across phenological stages, data from three weather stations associated with the breeding colonies were collected (Table 2). We used data from two Chilean navy weather stations: one in Paso Tortuoso, located 16.66 km from the Rupert and Monmouth colonies, and one in Puerto Harris (Dawson Island), located 20.41 km from Tuckers Islets. For the Contramaestre colony, we obtained meteorological data from a weather station on the island.

**TABLE 2:** Average ± standard deviation of temperature, precipitation, wind speed, and relative humidity across geographic regions in the Magellan Strait. We report the values for the first month of each phenological stage: courtship—October; egg‐laying—December, and chick‐rearing—February.

Phenological stage	Region^a^	Variables			
		Temperature (°C)	Precipitation (mm)	Wind speed (ms^−1^)	Relative humidity (%)
Courtship	Contramaestre	7.4 ± 2.1	0.005 ± 0.05	8.2 ± 3.9	74.6 ± 7.8
	Pacific	5.7 ± 2.3	0.204 ± 0.4	4.9 ± 5.3	81.8 ± 10.4
	Southern	7.8 ± 2.6	0.249 ± 1.2	9.1 ± 6.0	69.7 ± 11.9
Egg‐laying	Contramaestre	9.8 ± 2.4	0.014 ± 0.13	7.7 ± 3.6	71.7 ± 9.1
	Pacific	9.1 ± 2.5	No data	14.4 ± 6.1	74.5 ± 12.0
	Southern	10.6 ± 3.1	0.381 ± 1.8	10.0 ± 5.0	66.3 ± 13.8
Chick‐rearing	Contramaestre	10.5 ± 2.4	0.009 ± 0.07	7.8 ± 3.6	73.4 ± 7.4
	Pacific	8.7 ± 2.7	0.211 ± 0.4	3.4 ± 1.8	82.7 ± 9.3
	Southern	8.9 ± 2.5	0.071 ± 0.2	3.1 ± 2.3	78.9 ± 11.4

^a^ Region refers to the most proximal oceanic influence in the colonies. Pacific refers to the meteorological station located on Bahia Mussel, near the Rupert and Monmouth colonies. Southern refers to the meteorological station located on Dawson Island, near the Tuckers 1 and 2 colonies. Finally, Contramaestre refers to the meteorological station located on the Contramaestre colony.

In most cases, the data used to characterise environmental seasonality in breeding colonies were temperature, relative humidity, precipitation (but we could not obtain precipitation data from Paso Tortuoso weather station during November and December), and wind speed. Data were measured every 30 min in the weather station in Contramaestre during all day, and every 3 h in the AMCP‐FC and Tucker weather stations, from midday to midnight. Meteorological data during the chick‐rearing stage were taken during February, which was the month when data were available in all stations.

### 2.3 Animal Ethics

All penguin samples were collected following approved bioethical guidelines of the Comité de Ética, Bioética y Bioseguridad, Universidad de Concepción, Chile (protocol number CEBB 1081‐2021), and a sampling permit of the Subsecretaría de Pesca y Acuicultura, Chile (resolution E‐2021‐531).

### 2.4 Sample Collection, Storage and Processing

Microbial DNA was collected during the breeding season from penguins' feathers, nest soil, and seawater adjacent to each island colony. Sample collection took place during the breeding phenology of Magellanic penguins, defined as courtship (October), egg‐laying (December), and chick rearing (January) across the five islands previously described (Figure 1, sample details in Table 3), where a total of 206 samples was collected.

**TABLE 3:** Sampling characteristics of Magellanic penguin ( *Spheniscus magellanicus* ) plumage and associated environmental microbiota in the Magellan Strait, Chile.

Colony	Phenological stage (month)	Sample type	Sample size
Contramaestre	Courtship (October)	Penguins (chest and back feathers)	15
		Nest soil	7
	Chick‐rearing (January)	Penguins (chest and back feathers)	19
		Nest soil	9
		Water	1
Rupert	Courtship (October)	Penguins (flank swabs)	16
		Nest soil	15
	Egg‐laying (December)	Penguins (chest and back feathers)	9
		Nest soil	10
		Water	1
	Chick‐rearing (January)	Penguins (chest and back feathers)	8
		Nest soil	6
		Water	1
Monmouth	Egg‐laying (December)	Penguins (flank swabs)	6
		Water	1
	Chick‐rearing (January)	Penguins (chest and back feathers)	8
		Nest soil	5
Tuckers 1	Courtship (October)	Penguins (chest and back feathers)	10
		Nest soil	6
	Egg‐laying (December)	Penguins (chest and back feathers)	13
		Nest soil	1
		Water	1
	Chick‐rearing (January)	Penguins (chest and back feathers)	8
		Nest soil	4
		Water	1
Tuckers 2	Courtship (October)	Penguins (chest and back feathers)	6
		Nest soil	5
	Egg‐laying (December)	Penguins (chest and back feathers)	5
		Nest soil	2
	Chick‐rearing (January)	Penguins (chest and back feathers)	4
		Nest soil	3

*Note:* We did not sample the same individuals across breeding stages, since we did not have a permit to tag animals. All samples were kept to describe taxonomic patterns; however, to prevent spurious effects from sampling variability during statistical analyses, we removed penguin samples taken with swabs, those from Rupert during the courtship stage and those from Monmouth during the egg‐laying stage.

Penguin feather samples were obtained by directly cutting feathers or by swabbing feathers depending on if the penguin handling was feasible. The penguin handling procedure consisted of pushing the penguin from inside the burrow using a hook stick and carefully bringing the penguin outside the burrow for operators to immobilise it. The operator immobilising each penguin was wearing nitrile disposable gloves and a disposable plastic apron. Each penguin handling required two to three qualified operators wearing sterile nitrile disposable gloves to cut the tip of ca. 20 feathers (ca. 10 feathers from the back and ca. 10 feathers from the chest) using a sterile scalpel blade. After sampling, penguins were released into their nest. Penguin handling was not feasible when there was only one individual in the nest during egg incubation. In these cases, penguin feathers were sampled with swabs without taking penguins out of their burrow. A swabbing stick composed of three swabs pasted on one end of a stick was carefully introduced inside the nest to rub the feathers of the penguin. We did this in the Rupert colony during October and the Monmouth colony during December, since we found unexpectedly asynchronous laying eggs behaviour. Feather penguin samples were separately placed in 2 mL sterile cryotubes. Nest soil samples were taken using a 15 mL sterile falcon tube from the soil inside the nest. Feather and soil samples were kept at environmental temperature (cold) for approximately 3 h before being stored in liquid nitrogen upon arrival at the vessel. Seawater samples were taken from the vessel by filtering 3 L of seawater with a metal flute connected to a vacuum pump and polycarbonate membrane filters (0.2 μm pore size and 47 mm diameter, Advantec, MFS Inc.). Filters were placed in 2 mL cryotubes and immediately stored in liquid nitrogen. Upon arrival from the field, the samples were submitted to the CEQUA Molecular Genetics and Genomics Laboratory and kept at −80°C.

### 2.5 DNA Extraction and Sequencing

Samples were processed for DNA extraction between October 2022 and January 2023. Depending on penguin feather sample type, either all feathers or two swabs were considered for extraction. For nest soil samples, 100 mg of soil was weighed. For seawater samples, one filter was used. DNA extraction was conducted with the FastDNA SPIN Kit for Soil (MPBio, USA), following manufacturer instructions, with an initial incubation step at 56°C for 20 min, then agitation with Fast Prep at 6 m/s for 40 s.

Two procedures were introduced for quality control at this stage: blank negative control and designed mock communities. Blank negative controls were extracted to monitor the presence of reagent contamination. These failed to give the minimum DNA concentration to be sequenced. We also extracted DNA from four mock communities designed with the DNA of five species of *Bacillus* and one of *Domibacillus* from Cuatro Ciénegas, Mexico. These species were grown in a marine medium at 30°C for 24 h. The mock communities had the following variation: ‘compound’ mock (two), which consisted of the pooled DNA of the six species, and ‘integrated’ mock (two), which consisted of one extraction of the pooled tissue of all colonies. Hence, the expected taxonomic composition of these mock communities would contain mainly *Bacillus*, followed by *Domibacillus*. Mock communities were sequenced in different runs.

All DNA extractions were concentrated using a SpeedVac Thermo Savant (Thermo Scientific, model DNA 120‐230) for 30 min with standard conditions, which reduced volume to half (i.e., from 100 to 50 μL). Of this, 15 μL of DNA from each of the 206 biological samples, five DNA blank controls and the four mock communities were sent to Macrogen (Korea) for amplification of the hypervariable region V3–V4 of the 16S gene using the universal primers 341F‐805R, library preparation and paired‐end (2 × 300 bp) sequencing using Illumina MiSeq.

### 2.6 Inference of Amplicon Sequence Variants

To describe amplicon sequence variants (ASVs), raw sequences were processed in R v4.1.3 using DADA2 v1.18.0 (Callahan et al. 2016; R Core Team 2022). Primer sequences were trimmed in R. Forward and reverse reads were truncated at positions 290 and 250, respectively. Ambiguous bases were not allowed, and a maximum of two expected errors was set. Subsequent steps, including error rates learning, dereplication, denoising, and merging of paired reads, were performed using default parameters. Taxonomic assignment was performed with the naïve Bayesian classifier (Wang et al. 2007), using the Silva v138 database as a reference (Quast et al. 2013). Sequences assigned to chloroplasts and mitochondria and those detected in only one sample were discarded.

The resulting sequences were filtered with ‘decontam’, an open‐source R package that implements a statistical classification procedure to remove potential contaminant sequences from DNA extraction kits (Davis et al. 2018). Given that blank controls failed to give any sequence, potential contaminants were identified based on the final library DNA concentrations. Sequences whose abundance correlated inversely with DNA concentration were flagged as contaminants and filtered out (Davis et al. 2018). We detected 325 ASVs distributed in 57,837 sequences flagged as putative contaminants, which were filtered out. To prevent the influence of differences in sequencing depth in subsequent community diversity analyses, samples were normalised to the lowest sample size (McKnight et al. 2019), that is, 30,625 sequences, which yielded representative microbial communities in all colonies and sample types, according to rarefaction plots (Figure S1). After removing potential contaminant sequences and normalising sequencing depth, amplicon sequence data from all 206 biological samples was retained. The amplicon rarefied set yielded a total of 6,308,750 sequences, distributed in 67,673 ASVs.

### 2.7 Community Analysis

To visualise the top 10 most abundant bacterial genera, taxa were aggregated and graphed in heatmaps using the ‘microbiome’ R package (Lahti and Shetty 2017). In addition, to determine the core bacteria specific to penguin samples across phenological stages and colonies, the ‘core’ function of the microbiome package was used with a minimum prevalence of 90% and minimum relative abundance of 0.1%. Also, the taxonomic composition of mock communities was included.

To measure alpha diversity, the Shannon index was calculated with the R package phyloseq (McMurdie and Holmes 2013). Two statistical questions on alpha diversity data were performed: (1) Is phenology associated with changes in microbial alpha diversity in each island? and (2) Within each phenological stage, does penguin feather alpha diversity vary across colonies? We used the Kruskal–Wallis test to address these questions, using Sample type (whether it was penguin feather, nest soil or water sample) and Colony as categorical predictors, respectively. Significant results were further addressed to detect specific differences with the *post hoc* Wilcoxon ranks sum test, using Holm *p*‐value adjustment for multiple comparisons.

Two bacterial genera that could serve as biosensors of climate change progress in host‐microbial communities in the Magellan Strait were selected; *Psychrobacter*, a psychrophilic common bacterium in sub‐ and Antarctic ecosystems, and *Deinococcus*, a thermophilic, UV‐resistant bacterium. We compared the relative abundance of each genus in penguin feathers across phenological stages in each colony and within each phenological stage across colonies. A Bayesian approach was used to test if the relative abundance (response variable) of these selected genera changed across categorical predictors, using the ‘brms’ R package (Bürkner 2017a). We used beta regression since the response variable was a proportion (values bounded between 0 and 1). All models used default uninformative priors. These Bayesian Models use a Markov Chain Monte Carlo sampler, implemented with Rstan (Stan Development Team 2023), to estimate posterior distributions (Bürkner 2017b). Models were implemented with four parallel chains, each with 1000 warm‐up samples embedded in 4000 iterations. Chains convergence was diagnosed using Rhat values equal to 1, and bulk effective samples were equal or greater than 10% total posterior draws. In addition, posterior estimated parameters (mean and standard deviation) were checked to resemble those of the observed values (Gabry et al. 2019). The estimated mean and 95% credibility intervals were plotted within each breeding colony across phenological stages, and within each phenological stage across breeding colonies.

To determine differences in bacterial community structure (i.e., beta diversity) across sample types, within and across colonies, the Bray–Curtis (BC) index was used. Beta diversity was compared using the permutational analysis of variance (PERMANOVA) (Anderson 2017). Samples that lacked replicates were excluded from the test due to their lack of biological variability (i.e., water samples and nest soil in the Tuckers islet 1 colony during the egg‐laying stage). To detect specific significant differences between conditions, *post hoc* paired PERMANOVA tests with *p*‐value Holm adjustment for multiple comparisons were performed. To account for unbalanced sampling, the distance to the centroid within each factor was compared using the permutest function in the vegan package v2.6.4 (Oksanen et al. 2022) to judge the adequacy of PERMANOVA results (Anderson and Walsh 2013). We used a Bayesian model approach (as described above) to measure the effect of phenology and biogeography in the feather microbial similarity (BC distance − 1).

To determine the effect of environmental heterogeneity on the penguin feather and nest soil microbiota composition, Mantel tests were conducted to determine covariation between BC microbial distance and environmental distance. Given that we have only one measurement across each site, the average values of each available measurement (see Table 2 for abiotic factors considered) were used to construct the environmental distance matrix. Considering the abiotic environmental variation associated with seasonal changes, Mantel tests were performed in each phenological season.

Also, to test the effect of island size on microbial alpha diversity and the distance‐decay pattern, Spearman correlations were conducted between microbial alpha diversity and island size and penguin feather microbial compositional similarity (BC − 1) and island distance to the continent across each phenological stage, respectively.

Given the proximity of the colonies, we explored whether there was spatial autocorrelation among microbial alpha diversity and community similarity. We attempted to compute the Moran Index, but given the low number of replicates (i.e., colonies, from 3 to 5 depending on the phenological stage), the statistical coefficients computed were not stable. Hence, we did classical statistical comparisons (ANOVA followed by Tukey pairwise comparison) to detect similarities between the response variables across each factor. In general, we found that microbial alpha diversity was similar across stages, but it differed during the chick‐rearing stage (Tables S1–S4). In contrast, community similarity differed across colonies in each phenological stage, suggesting that despite the geographic closeness of the colonies, community similarity has significant variation (Tables S5–S10).

### 2.8 Environmental Data Analysis

To test for differences in temperature, relative humidity and wind speed across phenological stages, Kruskal tests followed by Wilcoxon pairwise tests were conducted to determine if there were differences between the selected environmental variables.

## 3 Results

### 3.1 Environmental Variables Across Geographic Regions and Phenological Stages

**FIGURE 2:**
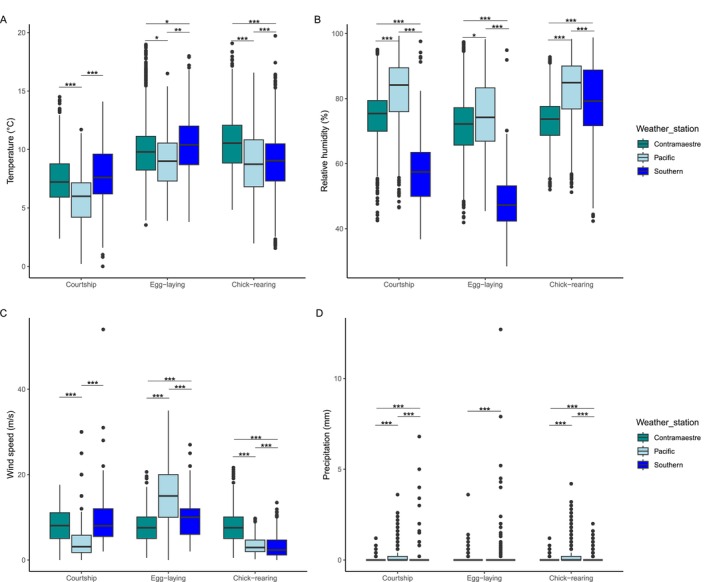
Environmental seasonality in the Magellan Strait across the breeding cycle of the Magellanic penguin. The weather station labelled as ‘Pacific’ is close to the Rupert and Monmouth colonies, whereas the one labelled as ‘Southern’ is close to the Tuckers 1 and Tuckers 2 colonies. (A) Temperature, (B) Relative humidity, (C) Wind speed and (D) Precipitation. Courtship stage values were measured during October, egg‐laying values were measured during December and chick‐rearing values were measured during February. The Kruskal–Wallis test was used to test the significance among weather stations in each phenological stage. Statistical notation: *p < 0.05, **p < 0.001, ***p < 0.0001. The boxplot depicts the first, second (median, black line) and third quartile, while the whiskers show interquartile range, and black dots indicate outliers.

All environmental measures considered differed across colonies in each phenological stage (Table 2, Figure 2, temperature, KW = 2113.3, df = 8, *p*‐value < 0.001; relative humidity, KW = 2013.7, *p*‐value < 0.001; wind speed, KW = 3721, *p*‐value < 0.001; precipitation, KW = 1194.8, *p*‐value < 0.001). *Post hoc* tests revealed that most environmental factors differ between geographic regions within each phenological stage. However, temperature and wind speed were similar during the courtship stage between the Contramaestre colony and the Tuckers islets colonies (*p*.adjusted = 0.072 and 1, respectively).

The environmental conditions that penguins experience across the colonies considered in this study follow complex and dynamic patterns across the austral seasonality. In the Contramaestre colony, penguins consistently experience the lowest precipitation across phenological stages. Also, during the chick‐rearing stage, the Contramaestre colony experiences the highest windspeed and temperature (Figure 2, Table 2). In contrast, penguins in the Rupert and Monmouth colonies (Pacific region) consistently experience the lowest temperatures and highest precipitation and relative humidity (Figure 2, Table 2). Finally, penguins in the Tuckers 1 and 2 colonies (Southern region) experience intermediate values of temperature and precipitation in relation to the Contramaestre colony and the Pacific region (Figure 2, Table 2).

### 3.2 Taxonomic Landscape in Penguin Feather, Nest Soil, and Seawater Microbiota

The taxonomic composition at the family level followed stable patterns across phenological and biogeographical factors (Figure S2). In most colonies and phenological stages, the most abundant bacterial family associated with penguin plumage was *Moraxellaceae* (Figure S2A–E). However, in the Monmouth colony, *Flavobacteriaceae* was the most abundant family in the feathers of penguins (Figure S2C). In contrast, the most abundant bacterial families in nest soil microbiota were *Micrococcaceae* in the Contramaestre colony (Figure S2A) and *Flavobacteriaceae* in the other nesting colonies (Figure S2B–E). Seawater microbiota adjacent to the nesting colonies were characterised by high abundances of *Moraxellaceae* in Contramaestre Island colony (Figure S2A) and *Flavobacteriaceae* in Monmouth, Rupert, and Tuckers islet 2 colonies (Figure S2B–E). As expected, the taxonomic composition of the mock communities exclusively consisted of *Bacillus* and *Domibacillus* (Figure S3).

### 3.3 Penguin‐Associated and Nest Soil Core Bacteria Landscape Across Colonies and Phenological Stages

We found a set of several bacterial genera with a minimum relative abundance of 0.001 shared among either 90% of penguin samples or nest soil samples across all nesting colonies and phenological stages. Penguin feather core genera are *Arthrobacter*, *Pedobacter*, *Psychrobacter*, *Pseudoarthrobacter*, *Nocardioides*, *Sporosarcina,* and *Clostridium sensu stricto 1* (Figure S4). Whereas nest soil core genera are *Tomitella*, *Arthrobacter*, *Flavobacterium*, *Pedobacter,* and *Sporosarcina*. *Psychrobacter* was the most abundant core genus in penguin samples across colonies and phenological stages.

### 3.4 Phenological Patterns in Penguin Feather Microbial Traits

**FIGURE 3:**
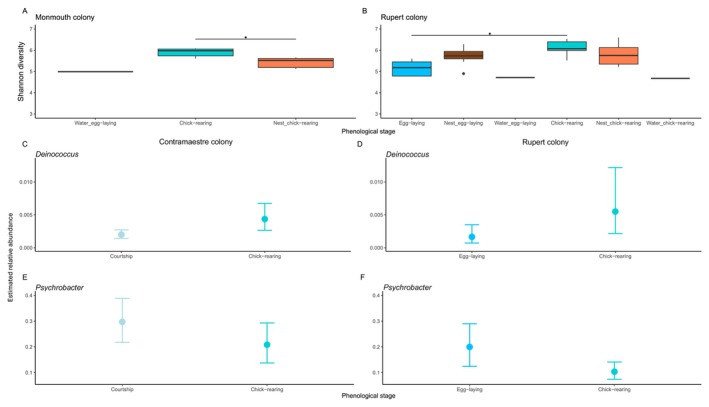
Phenology patterns in the penguin feather alpha diversity and the relative abundance of some genera in some colonies. Phenology is associated with changes in microbial alpha diversity from sample types: (A) In the Monmouth colony during the chick‐rearing stage, penguin feathers have higher diversity than nest soil; (B) In the Rupert colony, penguin feathers increase their alpha diversity from the egg‐laying to the chick‐rearing stage. (C, D) Phenological patterns in the relative abundance of *Deinococcus* bacteria from the penguin feather microbiota in the Contramaestre colony (C) and the Rupert colony (D). (E, F) Phenological patterns in the relative abundance of *Psychrobacter* bacteria from the penguin feather microbiota in the Contramaestre colony (E) and the Rupert colony (F). From panels (C–F), dots show estimated average relative abundance and lines represent 95% credibility intervals.

We detected similar microbial alpha diversity in the feather and nest soil samples across courtship, egg‐laying, and chick‐rearing stages in the Contramaestre, Tuckers 1, and Tuckers 2 colonies (Figure S5). In contrast, in the Monmouth and Rupert nesting colonies, the Shannon diversity of the feather microbiota differed across sample types and phenological stages (Figure 3A, KW = 8.167, *p* = 0.013; Figure 3B, KW = 19.749, *p* = 0.001, respectively). Wilcoxon *post hoc* test revealed that Shannon diversity differed between penguin feathers and nest soil during the chick‐rearing stage in Monmouth (*p*.adjusted = 0.019) and in penguin feathers from the egg‐laying to the chick‐rearing stage in Rupert (*p*.adjusted = 0.014) nesting colonies.

Phenology had a dynamic effect on the relative abundance of *Psychrobacter* and *Deinococcus* in the feather microbiota across nesting colonies. A phenological‐associated increase in the average relative abundance of *Deinococcus* was detected in the Contramaestre nesting colony (Figure 3C), with an increase of 0.24% (from 0.19% to 0.43%). In the Rupert nesting colony (Figure 3D) there was an increase of 0.38% from the egg‐laying (0.16%) to the chick‐rearing stage (0.55%). In contrast, the average relative abundance of *Psychrobacter* decreased across phenological stages. In the Contramaestre nesting colony, it decreased by 8.8% (Figure 3E, from 29.6% to 20.8%). Finally, in the Rupert nesting colony (Figure 3F), it decreased by 9.6% from the egg‐laying (20%) to the chick‐rearing stage (10.4%).

In contrast, in the Tuckers nesting colonies, the average relative abundance of *Deinococcus* in penguin feather microbiota was relatively similar across phenological stages. In the Tuckers 1 and 2 colonies, subtle dynamic variation below 1% was detected across phenological stages (Figure S6A,B). In contrast, the average relative abundance of *Psychrobacter* in the Tuckers colonies had a dynamic pattern, where it increased from the courtship to the egg‐laying stage and then decreased from the egg‐laying to the chick‐rearing stage (Figure S6C,D).

### 3.5 Geographic Patterns in Penguin Feather Microbial Traits

**FIGURE 4:**
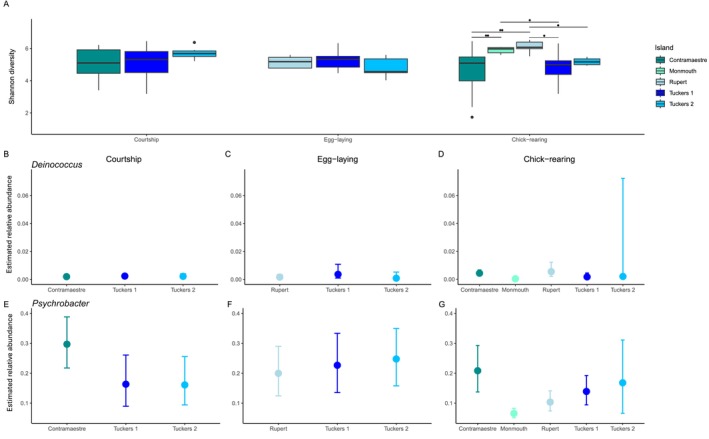
Geographic patterns in the penguin feather alpha diversity and the relative abundance of *Deinococcocus* and *Psychrobacter*. (A) Geographic patterns of penguin feather alpha diversity within each phenological stage. (B–D) The geographic pattern in the relative abundance of *Deinococcou*s in the feather microbiota of penguins during (B) courtship, (C) egg‐laying and (D) chick‐rearing stages. (E–G) The geographic pattern in the relative abundance of *Psychrobacter* in the feather microbiota of penguins during (E) courtship, (F) egg‐laying and (G) chick‐rearing stages. Dots show estimated average relative abundance, and lines represent 95% credibility intervals.

Penguin feather microbiota alpha diversity (Shannon index) was similar across all nesting colonies during the courtship (Figure 4A, KW = 1.701, *p* = 0.427) and egg‐laying stages (Figure 4A, KW = 1.588, *p* = 0.451). However, there were significant differences during the chick‐rearing stage (Figure 4A, Shannon, KW = 23.020, *p* < 0.001). The Wilcoxon *post hoc* test indicated that penguins from Rupert and Monmouth nesting colonies had the highest alpha diversity in their plumage. Particularly, the Shannon diversity from the feather microbiota in the Rupert nesting colony was higher than its equivalent from to the Contramaestre (*p*.adjusted = 0.006) and Tuckers 1 (*p*.adjusted = 0.043) nesting colonies. In turn, Shannon diversity in the penguin feather microbiota in the Monmouth nesting colony was higher compared to those in Contramaestre (*p*.adjusted = 0.006), while it was highly variable from penguins in the Tuckers 1 (*p*.adjusted = 0.037) and Tuckers 2 (*p*.adjusted = 0.059) nesting colonies. We also explored whether there was a significant correlation between island size and alpha diversity (richness and Shannon diversity) in the feather microbiota, yet we did not detect any significant association in any phenological stage (Figure S7).

The geographic location of the nesting colony influenced the relative abundance of *Deinococcus* and *Psychrobacter*. During the courtship stage (Figure 4B), penguins from the Tuckers 1 colony harboured the greatest proportion of *Deinococcus* (0.24%), followed by penguins from Tuckers 2 (0.22%), and Contramaestre (0.19%) nesting colonies. In turn, during the egg‐laying stage (Figure 4C), *Deinococcus* had a higher relative abundance in penguins from Tuckers 1 (0.36%), followed by those from Rupert (0.16%), and Tuckers 2 (0.08%) nesting colonies. Finally, in the chick‐rearing stage (Figure 4D), *Deinococcus* had a higher relative abundance in penguins from Rupert (0.50%), followed by penguins from Contramaestre (0.43%), Tuckers 2 (0.20%), Tuckers 1 (0.17%), and Monmouth (0.03%) nesting colonies. However, the average estimated proportion of *Deinococcus* is under more uncertainty in some colonies and phenological stages, as 95% credibility intervals are wider (hence the estimated average proportion is more uncertain) during the egg‐laying stage in Tuckers colonies (1 and 2) and in the chick‐rearing stage in Tuckers 2.

The relative abundance of *Psychrobacter* in the feather microbiota also varied across the geographic location of the nesting colony in each phenological stage. During the courtship stage (Figure 4E), feather microbiota from penguins in the Contramaestre colony harboured the greatest relative abundance of *Psychrobacter* (29.7%), followed by penguins from Tuckers 1 (16.3%), and Tuckers 2 (16.1%) colonies. In turn, during the egg‐laying stage (Figure 4F), *Psychrobacter* had a higher relative abundance in the feather microbiota of penguins from Tuckers 2 (24.8%), followed by penguins from Tuckers 1 (22.7%), and Rupert (20%) nesting colonies. Finally, in the chick‐rearing stage (Figure 4G), *Psychrobacter* had a higher relative abundance in penguins from Contramaestre (20.8%), followed by penguins from Tuckers 2 (16.8%), Tuckers 1 (13.9%), Rupert (10.5%), and Monmouth (6.57%) nesting colonies. As in *Deinococcus*, the average estimated relative abundance of *Psychrobacter* is under more uncertainty in some colonies and phenological stages; specifically, the credibility interval is wider during the chick‐rearing stage in Tuckers 2.

### 3.6 Phenological and Geographical Penguin Feather Microbiota Compositional Landscapes

**FIGURE 5:**
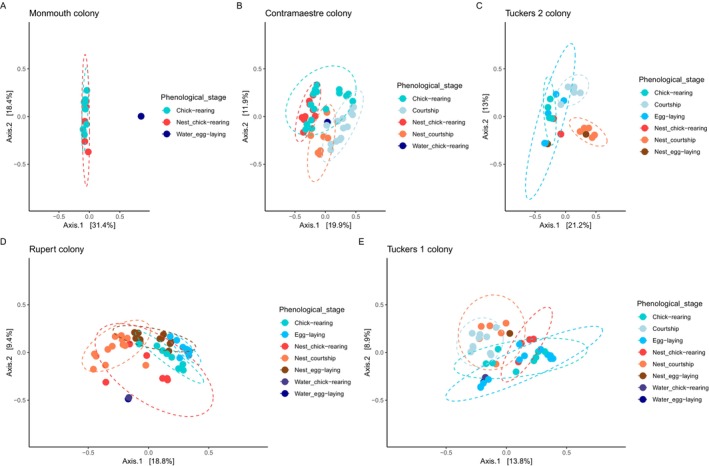
Ecological ordination of Magellanic penguin plumage microbiota across colonies and phenological stages. Dotted ellipses represent the 95% confidence region of the dispersion cloud of each factor level; each dot is a sample. Bray–Curtis based ordination of (A) Monmouth, (B) Contramaestre, (C) Tuckers 2, (D) Rupert and (E) Tuckers 1 colonies.

Phenology consistently explains microbial compositional differences between feather and nest soil microbiota within each nesting colony; Rupert (Figure 5A, pseudo‐*F* = 5.008, *R* ^2^ = 0.301, *p* = 0.001), Tuckers 1 (Figure 5B, pseudo‐*F* = 2.941, *R* ^2^ = 0.246, *p* = 0.001), Tuckers 2 (Figure 5C, pseudo‐*F* = 3.092, *R* ^2^ = 0.448, *p* = 0.001), Monmouth (Figure 5D, pseudo‐*F* = 2.968, *R* ^2^ = 0.270, *p* = 0.001), and Contramaestre (pseudo‐*F* = 5.234, *R* ^2^ = 0.254, *p* = 0.001).

PERMANOVA paired comparisons revealed that feather and nest soil harboured different microbial compositions across each phenological stage in several nesting colonies (Tables S11 and S12), although similar microbial compositions were detected between nest soil and penguin plumage on the Tuckers 2 nesting colony in December (pseudo‐*F* = 1.167, *R* ^2^ = 0.189, *p*.adjusted = 0.328), and between nest soil of October and December (pseudo‐*F* = 1.404, *R* ^2^ = 0.219, *p*.adjusted = 0.207), and December and January (pseudo‐*F* = 1.681, *R* ^2^ = 0.359, *p*.adjusted = 0.321).

Microbial dispersion was similar among most nesting colonies, either with the complete set (Tuckers 2 colony, *F* = 0.717, *p* = 0.657) or when removing factor levels that only had one sample (Contramaestre colony without water, *F* = 0.928, *p* = 0.434; Monmouth colony without water, *F* = 1.294, *p* = 0.297; Tuckers 1 colony without water and nest soil in the egg‐laying stage, *F* = 0.995, *p* = 0.406). However, in the Rupert colony, despite removing water, the differences in microbial compositional dispersion across conditions remained (*F* = 6.326, *p* = 0.001). Hence, in most nesting colonies, differences in the PERMANOVA test reflect microbial compositional differences, but in the Rupert colony, microbial compositional differences are confounded by compositional dispersion heterogeneity.

The relationship between phenology and feather microbial similarity (BC distance − 1) was variable among colonies (Figure S8). In the Contramaestre colony, feather microbial similarity decreased as phenology progressed (Figure S8A). In the Rupert and Tuckers 2 colonies (Figure S8C,E), feather microbial diversity had a dynamic pattern across phenological stages, where it either increased or decreased after each phenological stage. Finally, in the Monmouth and Tuckers 1 colonies, feather microbial similarity consistently increased as phenology progressed (Figure S8B,D).

### 3.7 Penguin Feather Microbiota Composition Exhibits Biogeographic Signals That Follow Their Colony Distribution

**FIGURE 6:**
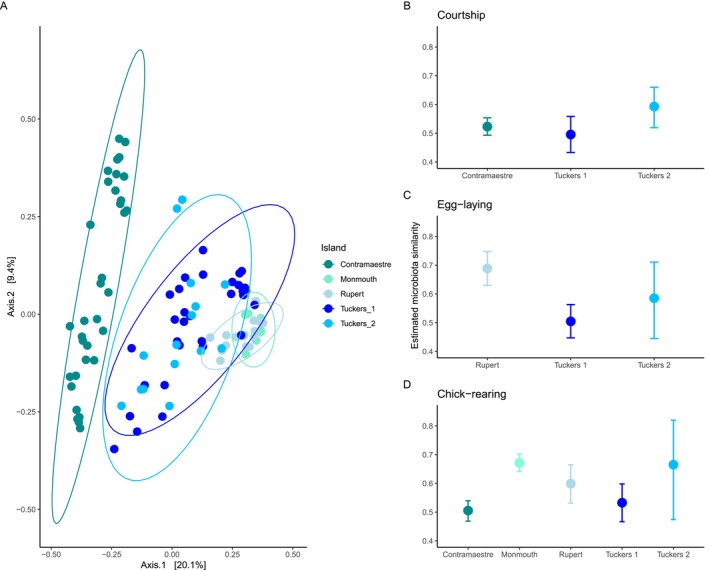
Geographic patterns in the feather microbiota composition and similarity. (A) Ecological ordination in the feather microbiota of Magellanic penguins. Geographic patterns in the estimated microbiota similarity of penguin feathers during (B) courtship, (C) egg‐laying, and (D) chick‐rearing stages. Dots show estimated average similarity based on the Bray–Curtis distance, and lines represent 95% credibility intervals.

Despite microbial compositional changes associated with phenology, when considering all samples from each nesting colony, microbial compositional differences across colonies in the penguin feather microbiota were also detected (pseudo‐*F* = 8.135, *R* ^2^ = 0.210, *p* = 0.001; Figure 6A) as well as in the nest soil microbiota (pseudo‐*F* = 4.707, *R* ^2^ = 0.216, *p* = 0.001; Figure S9). Further PERMANOVA paired comparisons revealed that penguin plumage and nest soil microbiota composition were colony‐specific (Table S13). However, microbial dispersion was different between penguin feathers (*F* = 14.264, *p* = 0.001) and the nest soil microbiota (*F* = 8.028, *p* < 0.001). Hence, the PERMANOVA results are also influenced by differences in dispersion among the factor levels in both feather and nest soil microbiota.

The average feather microbial similarity varied across the geographic location of the nesting colonies in each phenological stage. During the courtship stage (Figure 6B), penguins from the Tuckers 2 colony had the greatest feather microbial similarity among them (59.3%), followed by penguins from Contramaestre (52.3%), Rupert (51.7%), and Tuckers 1 (49.6%) colonies. During the egg‐laying stage (Figure 6C), penguins from the nesting colonies of Rupert and Monmouth colonies had the highest feather microbial similarity (Rupert: 68.9%, Monmouth: 60.4%). In contrast, penguins from both Tuckers islets exhibited less microbial similarity during this stage, particularly those from Tuckers 1 (50.4%) and Tuckers 2 (58.5%). Finally, in the chick‐rearing stage (Figure 6D), penguins from the Monmouth colony had the greatest feather microbial similarity (67.1%), followed by penguins from Tuckers 2 (66.5%), Rupert (60%), Tuckers 1 (53.3%), and Contramaestre (50.5%).

We found a significant covariation between environmental conditions and penguin feather microbial distance across phenological stages: courtship (Mantel statistic *r*: 0.4103, *p* = 0.0001), egg‐laying (Mantel statistic *r*: 0.1685, *p* = 0.0011), and chick‐rearing (Mantel statistic *r*: 0.7806, *p* = 0.0001). Also, a significant covariation between the environmental conditions and nest soil microbial distance across phenological stages was detected: courtship (Mantel statistic *r*: 0.1611, *p* = 0.0029), egg‐laying (Mantel statistic *r*: 0.4623, *p* = 0.0289), and chick‐rearing (Mantel statistic *r*: 0.6678, *p* = 0.0001), indicating a relationship between penguin feathers and nest soil microbiota composition and the environmental characteristics of their colonies.

**FIGURE 7:**
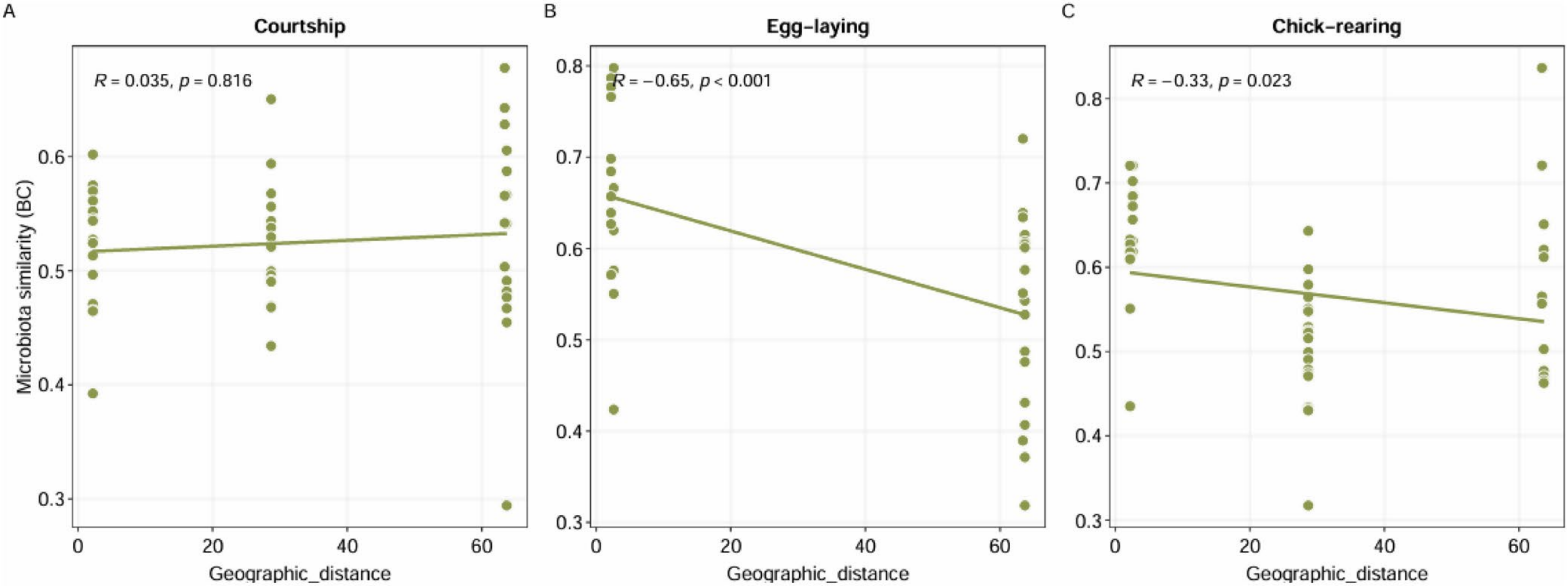
The distance‐decay pattern in penguin feather microbiota similarity. Each point represents one sample and each column of points represents the penguins corresponding to each colony according to its distance to the continent. Correlations between geographic distance to the continent (measured in kilometres) and feather microbiota similarity across phenological stages: (A) courtship, (B) egg‐laying stage and (C) chick‐rearing stage.

Contrasting relationships between geographic distance to the continent and penguin feather microbial similarity were found across phenological stages. In the courtship stage, there was no significant correlation between geographic distance (Figure 7A, *R* = 0.035, *p* = 0.816) and feather microbial similarity. However, in the egg‐laying and chick‐rearing stages, significant correlations were detected. Feather microbial similarity was negatively correlated with geographic distance in the egg‐laying (Figure 7B, *R* = −0.65, *p* < 0.001) and the chick‐rearing (Figure 7C, *R* = −0.33, *p* = 0.023) stages.

## 4 Discussion

We characterised the feather microbiota of Magellanic penguins across their reproductive cycle in five breeding colonies with contrasting environmental conditions in the Magellan Strait, Chile. Besides the reported effect that seasonality/phenology has on the gut and skin microbiota of vertebrates (Van Cise et al. 2020; Tong et al. 2020; Li, Li, et al. 2023), our work illustrates the joint effects of seasonality and geography on the feather microbial community at different levels.

### 4.1 Environmental Seasonality in the Magellan Strait

Penguin nesting colonies in the Magellan Strait experience strong seasonality involving changes in temperature, relative humidity, wind speed, and precipitation. In the Contramaestre colony, penguins experience the lowest precipitation across phenological stages, and during the chick‐rearing stage experience the greatest temperatures and wind speed. In contrast, penguins in the Monmouth and Rupert colonies experience the greatest precipitation, relative humidity and the lowest temperatures across phenological stages. Finally, penguins in the Tuckers 1 and 2 colonies experienced the highest temperatures and lowest relative humidity during the courtship and egg‐laying stages. We propose that this abiotic seasonality could have important consequences on their environmental microbiota, which in turn could influence the bacteria colonising penguin feathers. Changes in precipitation and relative humidity have a positive relation with alpha diversity and relative abundance of dominant taxa in soil microbiota (Zhao et al. 2022; Varliero et al. 2024). Seasonal increases in temperature and seasonal variability in humidity and precipitation could affect the relative abundance of psychrophilic bacteria which could affect the feather microbial diversity in penguin feathers (Oh et al. 2022). Our results show the dynamic abiotic conditions that Magellanic penguins experience across their colonies in the Magellan Strait, and how these abiotic conditions change throughout their phenological cycle.

### 4.2 Core Bacterial Taxa at the Landscape Level

Core genera that persist in most penguin feathers despite geographic and phenological differences include taxa like *Psychrobacter*, *Arthrobacter*, *Pedobacter*, *Pseudoarthrobacter*, *Nocardioides*, *Sporosarcina,* and *Clostridium sensu stricto 1*. The high prevalence of these bacterial genera across different nesting colonies and phenological stages could indicate a constant sourcing from environmental microbial relatives. *Psychrobacter* is the most abundant and prevalent taxon in the body surface of Magellanic and king penguins (Ochoa‐Sánchez, Acuña Gomez, Moreno, et al. 2023; Ochoa‐Sánchez, Acuña‐Gómez, et al. 2024), but is also common in the skin of marine mammals in subantarctic and Antarctic ecosystems (reviewed in Ochoa‐Sánchez, Acuña Gomez, Ramírez‐Fenández, et al. 2023). Interestingly, *Psychrobacter* is a highly abundant taxon in the blood from different penguin species (Ferchiou et al. 2025). Some of these penguin feather core genera may come from the nest soil, such as *Arthrobacter* and *Sporosarcina*, which are taxa with high prevalence in the nest soil microbiota despite temporal and geographical variability. Besides, penguin feather core genera are commonly found in glaciers and Antarctic soils, which could be plausible environmental sources (Lawson et al. 2000; Bowman 2006; Dsouza et al. 2015; Vega‐Celedón et al. 2021; Wang et al. 2023).

### 4.3 Phenology Affects Penguin Feather Alpha and Beta Diversity

We predicted that the effect of phenology on penguin feather microbiota alpha and beta diversity would be shaped by geography. We found different pieces of evidence supporting this prediction. While phenology only affected alpha diversity in the Rupert colony where it increased from the egg‐laying to the chick‐rearing stage, beta diversity consistently structured feather and nest soil microbial composition across colonies. Differences in feather microbial alpha diversity may be influenced by changes in environmental exposure due to behavioural changes associated with their breeding cycle. For example, during the egg‐lying stage, penguins experience low environmental exposure due to the prolonged periods they spend inside their nest incubating their egg. In contrast, in the chick‐rearing stage, penguins experience higher environmental exposure due to constant foraging to feed their chicks. This is in line with evidence from frog (Tong et al. 2020) and bat (Li, Li, et al. 2023) skin microbiotas, whose skin microbial alpha diversity increased from hibernation, when hosts were inactive and sheltered from the environment, to the active foraging stage when they had greater environmental exposure.

Consistent microbial compositional differences in feather and nest soil microbiota align with evidence from terrestrial and marine mammals, where skin microbiota composition was significantly structured by phenology (Tong et al. 2020; Li, Li, et al. 2023) and seasonality (Van Cise et al. 2020). Compositional changes remain relatively stable since community similarity stayed around 0.5 across phenological stages in each nesting colony. However, we detected different trends in feather microbial similarity as the phenological cycle progressed: decrease (Contramaestre), dynamic variation (Rupert and Tuckers 2) and increase (Tuckers 1 and Monmouth). Abiotic factors such as precipitation and temperature are important drivers of microbial communities and might underlie the different trends in feather microbial turnover in those colonies (Naidoo et al. 2022; Varliero et al. 2024). Also, the effect that environmental seasonality has on environmental microbial diversity could produce seasonal patterns in gut microbial‐enriched taxa (Baniel et al. 2021). Here, it could be possible that environmental seasonality produces a seasonal succession in microbial pools, exposing penguins to seasonally different microbial environmental pools, which affect feather and nest microbial composition and turnover across phenological stages.

### 4.4 Phenology Is Associated With Changes in the Relative Abundance of *Deinococcus* and *Psychrobacter*

Phenology influenced the relative abundance of *Deinococcus* and *Psychrobacter* in the feather microbiota, although in a colony‐specific way. The average relative abundance of *Deinococcus* subtly increased from the Courtship to the Chick‐rearing stage in the Contramaestre and Rupert nesting colonies. *Deinococcus* is an extremophile bacterium adapted to proliferate in arid conditions and under constant exposure to UV radiation (Liu et al. 2023). *Deinococcus* spp. are common bacteria in Antarctic soils (Hirsch et al. 2004), where they endure drastic environmental fluctuations and intense UV irradiation due to the ozone layer hole (Adlam et al. 2010). Interestingly, all colonies considered in this study might be exposed to higher levels of UV radiation, given the increments in the area and the persistence of anomalous holes in the ozone layer (Cordero et al. 2022). Penguin colonies along the Magellan Strait may be exposed to a richer pool of *Deinococcus* taxa given the atypical UV radiation that has been experienced in the region. Furthermore, colonies with dry conditions, such as the Contramaestre colony, might harbour a greater pool of *Deinococcus* taxa. Also, given the ephemeral nature of penguin feather microbiota composition (Ochoa‐Sánchez, Acevedo, et al. 2024), the load of *Deinococcus* taxa in the plumage may vary across daily activity patterns and behaviours.

The relative abundance of *Psychrobacter* decreased as the phenological cycle in the Contramaestre and Rupert nesting colonies. This pattern coincides with the seasonal increase in temperature in the chick‐rearing stage. The genus *Psychrobacter* has two ecotypes that differ in the width of their thermal tolerance: the stenothermal, which grows between 4°C and 25°C, and the eurythermal, which grows between 4°C and 37°C (Welter et al. 2021). Hence, it is plausible that the decrease in the relative abundance of *Psychrobacter* in these colonies reflects the seasonal loss of stenothermal taxa. The remaining *Psychrobacter* could be eurythermal taxa that persist despite thermic seasonality.

In contrast, the relative abundance of *Deinococcus* and *Psychrobacter* in the feather microbiota remained stable across phenological stages in the Tuckers 1 and 2 nesting colonies. The Tuckers colonies have different environmental features than the other colonies, particularly Tuckers 2, which is covered by a dense forest of *Nothofagus* trees that could buffer temperature seasonal changes (Feldman et al. 2023). Hence, penguins nesting in this colony might be under an environmental shelter that could buffer the effect that seasonality has on penguin feather microbiota. Under the ongoing climate change, it is expected that the relative abundance of psychrophilic taxa will decrease, whereas tropical taxa are expected to increase their range and colonise new polar ecosystems (Zarzyczny et al. 2024). Here, we predict that the increase in temperature in the next years could be related to the increase and decrease in the relative abundance of *Deinococcus* and *Psychrobacter*, respectively, in the surface microbiota of sub‐ and Antarctic hosts.

### 4.5 Geography Shapes Magellanic Penguin Feather Microbiota Traits

Geographic variability was associated with changes in feather microbial alpha and beta diversity. Feather microbial diversity was higher in the Rupert and Monmouth colonies, but only during the chick‐rearing stage. These colonies are the second and third biggest colonies in terms of total island surface, respectively. These results align with the Theory of Island Biogeography (MacArthur and Wilson 1967), which states that larger islands will have greater diversity. Island size correlates with higher environmental heterogeneity and ecological niches, universal drivers of microbial diversity across biomes (Stein et al. 2014; Huber et al. 2020). Also, precipitation and relative humidity are highest in the Monmouth and Rupert nesting colonies during the chick‐rearing stage. These abiotic factors, as well as variability in the vegetation cover, are associated with higher microbial alpha diversity (Zhao et al. 2022; Wong et al. 2023). Hence, environmental heterogeneity, coupled with higher precipitation and relative humidity, is associated with higher feather microbiota alpha diversity in these colonies during the chick‐rearing stage (i.e., austral summer).

The relative abundance of *Deinococcus* and *Psychrobacter* in the penguin feather microbiota follows different geographic patterns within each phenological stage. During the courtship and chick‐rearing stages, the highest relative abundance of *Deinococcus* occurred in the Rupert colony, while during the egg‐laying stage, it occurred in the Tuckers 1 colony. *Deinococcus* could be acquired by soil contact or wind dispersion (Hirsch et al. 2004). In turn, the highest relative abundance of *Psychrobacter* during the courtship and chick‐rearing stages occurred in the Contramaestre colony. During the egg‐laying stage, it occurred in the Tuckers 2 colony. The Contramaestre colony experiences the strongest winds during the courtship and chick‐rearing stages. Wind is an efficient medium to transport bacteria (Prospero et al. 2005; Smith et al. 2013), and *Psychrobacter* has been detected in bioaerosols (Bowers et al. 2009; Chen et al. 2021). Hence, wind may enhance *Psychrobacter* migration from environmental sources to the penguin plumage, particularly in the Contramaestre colony where no vegetation cover could limit the dispersion of air‐borne bacteria. Overall, the observed patterns highlight the relevance of dispersal limitation, ecological drift, Theory of Island Biogeography and environmental seasonality in the relative abundance of *Deinococcus* and *Psychrobacter* in the penguin plumage across its breeding cycle (Martiny et al. 2006).

Interestingly, penguin feather microbiota composition from each nesting colony follows the location of each colony in the Magellan Strait: the Atlantic group (Contramaestre colony), the Pacific group (Monmouth and Rupert colonies) and the Southern group (Tuckers 1 and 2 colonies). It would be interesting to include more colonies within these regions to validate these groups (e.g., the Magdalena nesting colony, neighbour to the Contramaestre nesting colony, should fall in the Atlantic group) or explore geographical patterns with different hosts to validate these groups. Moreover, feather microbial similarity was higher in colonies with dense vegetation cover (Rupert and Tuckers 2), while those with poor vegetation cover had lower microbial similarity (Contramaestre and Tuckers 1). This suggests that vegetation cover may modulate microbial turnover in the microbiota of penguins feathers, highlighting the role of environmental heterogeneity in feather microbial turnover (Li, Ma, et al. 2023). Further work with more hosts under ecosystems with different types of vegetation cover might aid in corroborating this pattern.

The distance‐decay pattern suggests a direct relationship between species turnover and island distance from the continent, resulting from the combined effects of differences in dispersal, colonisation potential, and ecological drift (MacArthur and Wilson 1967; Hubbell 2001; Leibold et al. 2004). We found significant covariation between feather microbial composition and environmental features, and a negative correlation between feather microbial similarity and geographic distance in the egg‐laying and chick‐rearing stages, indicating that feather microbial turnover increases as colonies are farther from the continent. Abiotic factors dynamically change across the breeding phenology of the Magellanic penguin, yet average temperature consistently increases from the courtship to the chick‐rearing stage. Temperature is a universal positive driver of the diversity of environmental microbiomes (Ward et al. 2017; Zhao et al. 2022). Also, given the different vegetation cover in the penguin colonies and its importance in the composition of the environmental microbiome (Bastida et al. 2021; Wong et al. 2023), it is plausible to conceive that environmental microbiomes experience idiosyncratic trajectories in their diversity and composition, which produce higher compositional heterogeneity in the penguin feather microbiota, ultimately leading to the distance‐decay pattern.

### 4.6 Relevance of Phenology and Geographic Variability in Wildlife Skin Microbiotas

Our results highlight how geographic variability modulates the effect of phenology in several traits of the feather microbiota in Magellanic penguins. Our results are of particular interest to hosts that have a wide distribution, such as this and other penguin species (Boersma and Borboroglu 2013), but also to several migratory marine animals, whose surface microbiome might be exposed to contrasting environmental conditions across their migration, such as other birds like petrels (Dias et al. 2011), marine mammals like humpback whales (Meynecke et al. 2021), and different fish groups (Block et al. 2005).

In our study, changes in microbial alpha diversity of the feathers were detected from the egg‐laying to the chick‐rearing stage, which appear to be contingent on the environmental features of the colony (size, precipitation, penguins, and relative humidity). Furthermore, consistent compositional shifts in the feather microbiota were detected across the breeding cycle in all colonies, extending to seabirds the known effect of phenology in wildlife microbiota of both terrestrial and marine hosts (Baniel et al. 2021; Tang et al. 2023; Tong et al. 2020; Van Cise et al. 2020; Li, Li, et al. 2023). Interestingly, feather microbiota composition in our study was influenced by the geographic region of the colony, which is consistent with differences in the climatic regime that these colonies experience. Hence, although our results indicate a consistent effect of phenology on the feather microbiota composition, feather microbiota traits recapitulate the distribution of the nesting colonies, likely as a consequence of the different environmental features that each colony faces.

Overall, our results highlight the importance of considering geographic variability when addressing the effect of phenology on a migratory animal, which might be valid for other hosts with a wide range of distribution. Ongoing work by our group is testing the effect of migration on humpback whales, which could serve to inform about the consistency of the results presented here.

## 5 Conclusions

Here, we characterised the feather microbiota of Magellanic penguins across five nesting colonies and three phenological stages: courtship, egg‐laying, and chick‐rearing, in the Magellan Strait, Chile. We found seven core bacterial genera in the penguin feather microbiota despite geographic and phenological variation. Phenology affected the feather microbiota alpha diversity in the Rupert nesting colony, where it increased from the egg‐laying to the chick‐rearing stage. Also, phenology produced consistent compositional shifts in the feather and nest soil microbiota across nesting colonies. Finally, phenology influenced the relative abundance of *Deinococcus* and *Psychrobacter* in the feather microbiota of most colonies. We found support for the predictions of the Theory of Island Biogeography in host‐associated microbial communities: (1) larger colonies had hosts with higher feather microbial diversity and (2) the distance‐decay pattern, that is, a significant correlation between feather microbiota similarity and colony distance to the continent. Finally, the relative abundance of *Deinococcus* and *Psychrobacter* was influenced by geographic variability. Our results highlight how temporal and environmental heterogeneity shape microbial traits in a marine host.

## Author Contributions

M.O.‐S. carried out conceptualisation, fieldwork, laboratory work, data curation, formal analyses and writing – original draft, review and editing. E.P.A.‐G. carried out fieldwork, conceptualisation, funding and writing – review and editing. C.A.M. carried out fieldwork and writing – review and editing. P.V. carried out fieldwork and writing – review and editing. J.A. carried out fieldwork and writing – review and editing. L.E.E. carried out conceptualisation, fieldwork and writing – review and editing. V.S. carried out conceptualisation, fieldwork, funding and writing – review and editing.

## Disclosure

Benefit‐Sharing Statement: This article confirms the fourth year of successful international collaboration between Instituto de Ecología (UNAM, México) and CEQUA (Chile). All collaborators are co‐authors. The results of this article belong to the ongoing Microbiome project (ANID project number R20F0009), which is spurring regional scientific development in southern Chile, led by a regional centre, CEQUA. As indicated above, all data have been shared with the broader public via appropriate public databases.

## Conflicts of Interest

The authors declare no conflicts of interest.

## Supporting information


**Appendix S1:** mec70114‐sup‐0001‐AppendixS1.docx.

## Data Availability

Raw 16S sequences were deposited in the NCBI under Bioproject numbers PRJNA1225745 (penguin feather samples) and PRJNA1225788 (environmental samples).
