# Supplementary material for: Feather Microbiota Landscapes: Biogeography and Phenology Shape Feather Microbiota Traits in a Migratory Seabird in a Subantarctic Ecosystem
Source: Mol Ecol. 2025 Oct 3;34(21):e70114. doi: 10.1111/mec.70114 (PMC12573722; doi:10.1111/mec.70114)
Supplement: Supplementary file 1 — Appendix S1: mec70114‐sup‐0001‐AppendixS1.docx. [file MEC-34-e70114-s001.docx]

**SUPPLEMENTARY MATERIAL**

**Feather microbiota landscapes: biogeography and phenology shape feather microbiota traits in a migratory seabird in a subantarctic ecosystem**

Authors: Manuel Ochoa-Sánchez*^1,2,3^, E. Paola Acuña-Gómez ^2^, Claudio A. Moraga^2^, Jorge Acevedo^2^, Pedro Valenzuela^2^, Luis E. Eguiarte^1^, Valeria Souza*^1,2^

^1^ Instituto de Ecología, Universidad Nacional Autónoma de México, CDMX, México

^2^ Centro de Estudios del Cuaternario de Fuego, Patagonia y Antártica (CEQUA), Punta Arenas, Chile

^3^ Posgrado en Ciencias Biológicas, Unidad de Posgrado, Edificio D, 1° Piso, Circuito de Posgrados, Ciudad Universitaria, Coyoacán, C.P. 04510, CDMX Universidad Nacional Autónoma de México, México

*Corresponding Authors:

Manuel Ochoa-Sánchez^1,2,3^

Valeria Souza^1,2^

Email address: manuel.ochoa@iecologia.unam.mx, souza@unam.mx

Supplementary Table 1. Shannon One-way ANOVA in the Courtship stage

|  | Df | Sum Sq | Mean Sq | F value | Pr(>F) |
| --- | --- | --- | --- | --- | --- |
| Island | 2.000 | 1.593 | 0.796 | 1.055 | 0.361 |
| Residuals | 28.000 | 21.127 | 0.755 |  |  |

Supplementary Table 2. Shannon One-way ANOVA in the Egg-laying stage

|  | Df | Sum Sq | Mean Sq | F value | Pr(>F) |
| --- | --- | --- | --- | --- | --- |
| Island | 2.000 | 0.746 | 0.373 | 1.433 | 0.258 |
| Residuals | 24.000 | 6.241 | 0.260 |  |  |

Supplementary Table 3. Shannon One-way ANOVA in the Chick-rearing stage

|  | Df | Sum Sq | Mean Sq | F value | Pr(>F) |
| --- | --- | --- | --- | --- | --- |
| Island | 4.000 | 19.259 | 4.815 | 5.561 | 0.001* |
| Residuals | 42.000 | 36.366 | 0.866 |  |  |

Supplementary Table 4. Tukey HSD *Post-hoc* Comparisons for Shannon One-way ANOVA in the Chick-rearing stage

| Comparison | Difference | Lower CI | Upper CI | Adjusted p-value |
| --- | --- | --- | --- | --- |
| Monmouth-Contramaestre | 1.3348 | 0.2172 | 2.4525 | 0.0122 |
| Rupert-Contramaestre | 1.5428 | 0.4252 | 2.6605 | 0.0027* |
| Tuckers 1-Contramaestre | 0.2467 | -0.8709 | 1.3644 | 0.9695 |
| Tuckers 2-Contramaestre | 0.6187 | -0.8401 | 2.0775 | 0.7464 |
| Rupert-Monmouth | 0.2080 | -1.1179 | 1.5339 | 0.9914 |
| Tuckers 1-Monmouth | -1.0881 | -2.4140 | 0.2378 | 0.1531 |
| Tuckers 2-Monmouth | -0.7161 | -2.3400 | 0.9078 | 0.7185 |
| Tuckers 1-Rupert | -1.2961 | -2.6220 | 0.0298 | 0.0582* |
| Tuckers 2-Rupert | -0.9241 | -2.5480 | 0.6998 | 0.4923 |
| Tuckers 2-Tuckers 1 | 0.3720 | -1.2519 | 1.9959 | 0.9652 |

Supplementary Table 5. Microbial community similarity One-way ANOVA in the Courtship stage

|  | Df | Sum Sq | Mean Sq | F value | Pr(>F) |
| --- | --- | --- | --- | --- | --- |
| Island | 2 | 0.036 | 0.018 | 3.69 | 0.038* |
| Residuals | 28 | 0.136 | 0.005 |  |  |

Supplementary Table 6. Microbial community similarity One-way ANOVA in the Egg-laying stage

|  | Df | Sum Sq | Mean Sq | F value | Pr(>F) |
| --- | --- | --- | --- | --- | --- |
| Island | 2 | 0.181 | 0.091 | 9.655 | 0.001* |
| Residuals | 24 | 0.225 | 0.009 |  |  |

Supplementary Table 7. Microbial community similarity One-way ANOVA in the Chick-rearing stage

|  | Df | Sum Sq | Mean Sq | F value | Pr(>F) |
| --- | --- | --- | --- | --- | --- |
| Island | 4 | 0.22 | 0.055 | 9.231 | 0* |
| Residuals | 42 | 0.25 | 0.006 |  |  |

Supplementary Table 8. Tukey HSD *Post-hoc* comparisons for the microbial community similarity One-way ANOVA in the Courtship stage

| Comparison | Difference | Lower CI | Upper CI | Adjusted p-value |
| --- | --- | --- | --- | --- |
| Tuckers 1-Contramaestre | -0.0266 | -0.0968 | 0.0437 | 0.6229 |
| Tuckers 2-Contramaestre | 0.0700 | -0.0131 | 0.1532 | 0.1114 |
| Tuckers 2-Tuckers 1 | 0.0966 | 0.0077 | 0.1855 | 0.0311* |

Supplementary Table 9. Tukey HSD Post-hoc comparisons for the microbial community similarity One-way ANOVA in the Egg-laying stage

| Comparison | Difference | Lower CI | Upper CI | Adjusted p-value |
| --- | --- | --- | --- | --- |
| Tuckers 1-Rupert | -0.1845 | -0.2894 | -0.0796 | 0.0006* |
| Tuckers 2-Rupert | -0.1028 | -0.2377 | 0.0321 | 0.1596 |
| Tuckers 2-Tuckers 1 | 0.0817 | -0.0456 | 0.2090 | 0.2639 |

Supplementary Table 10. Tukey HSD Post-hoc comparisons for the microbial community similarity One-way ANOVA in the Chick-rearing stage

| Comparison | Difference | Lower CI | Upper CI | Adjusted p-value |
| --- | --- | --- | --- | --- |
| Monmouth-Contramaestre | 0.1661 | 0.0734 | 0.2588 | 0.0001* |
| Rupert-Contramaestre | 0.0953 | 0.0026 | 0.1880 | 0.0413* |
| Tuckers 1-Contramaestre | 0.0275 | -0.0652 | 0.1202 | 0.9148 |
| Tuckers 2-Contramaestre | 0.1645 | 0.0435 | 0.2855 | 0.0032* |
| Rupert-Monmouth | -0.0708 | -0.1808 | 0.0392 | 0.3684 |
| Tuckers 1-Monmouth | -0.1386 | -0.2486 | -0.0286 | 0.0073* |
| Tuckers 2-Monmouth | -0.0016 | -0.1363 | 0.1331 | 1.0000 |
| Tuckers 1-Rupert | -0.0678 | -0.1778 | 0.0422 | 0.4117 |
| Tuckers 2-Rupert | 0.0692 | -0.0655 | 0.2039 | 0.5912 |
| Tuckers 2-Tuckers 1 | 0.1370 | 0.0023 | 0.2717 | 0.0446* |

Supplemental Table 11. Paired PERMANOVA statistics per colony across phenological stages. Light gray shade indicates non-significant comparisons.

| Colony | Sample type | Comparison | Pseudo-F | R^2^ | p adjusted |
| --- | --- | --- | --- | --- | --- |
| Contramaestre | Feathers | Courtship – Chick-rearing | 6.726 | 0.173 | 0.001 |
|  | Nest soil | Courtship – Chick-rearing | 3.139 | 0.183 | 0.001 |
| Rupert | Feathers | Courtship – Egg-laying | 8.635 | 0.272 | 0.001 |
|  |  | Egg-laying – Chick-rearing | 4.559 | 0.233 | 0.002 |
|  |  | Courtship – Chick-rearing | 4.583 | 0.172 | 0.001 |
|  | Nest soil | Courtship – Egg-laying | 3.998 | 0.148 | 0.001 |
|  |  | Egg-laying – Chick-rearing | 3.004 | 0.176 | 0.001 |
|  |  | Courtship – Chick-rearing | 2.896 | 0.132 | 0.003 |
| Monmouth | Feathers | Egg-laying – Chick-rearing | 3.281 | 0.214 | 0.003 |
| Tuckers 1 | Feathers | Courtship – Egg-laying | 4.507 | 0.176 | 0.003 |
|  |  | Egg-laying – Chick-rearing | 2.347 | 0.109 | 0.020 |
|  |  | Courtship – Chick-rearing | 3.095 | 0.162 | 0.003 |
|  | Nest soil | Courtship – Chick-rearing | 1.928 | 0.194 | 0.008 |
| Tuckers 2 | Feathers | Courtship – Egg-laying | 3.299 | 0.268 | 0.025 |
|  |  | Egg-laying – Chick-rearing | 2.385 | 0.254 | 0.049 |
|  |  | Courtship – Chick-rearing | 4.095 | 0.338 | 0.025 |
|  | Nest soil | Courtship – Egg-laying | 1.404 | 0.219 | 0.207 |
|  |  | Egg-laying – Chick-rearing | 1.681 | 0.359 | 0.321 |
|  |  | Courtship – Chick-rearing | 3.397 | 0.361 | 0.038 |

Supplemental Table 12. Paired PERMANOVA statistics per colony between penguin feathers and nest soil across each phenological stage. Light gray shade indicates non-significant comparisons.

| Colony | Phenological stage | Pseudo-F | R^2^ | p adjusted |
| --- | --- | --- | --- | --- |
| Contramaestre | Courtship | 4.262 | 0.175 | 0.001 |
|  | Chick-rearing | 3.270 | 0.111 | 0.004 |
| Monmouth | Chick-rearing | 2.044 | 0.156 | 0.025 |
| Rupert | Courtship | 4.628 | 0.137 | 0.001 |
|  | Egg-laying | 4.433 | 0.206 | 0.001 |
|  | Chick-rearing | 2.466 | 0.170 | 0.006 |
| Tuckers 1 | Courtship | 2.079 | 0.129 | 0.003 |
|  | Chick-rearing | 1.789 | 0.151 | 0.024 |
| Tuckers 2 | Courtship | 3.465 | 0.278 | 0.025 |
|  | Egg-laying | 1.167 | 0.189 | 0.328 |
|  | Chick-rearing | 2.700 | 0.350 | 0.049 |

Supplemental Table 13. Paired PERMANOVA statistics across breeding colonies.

| Colony | Sample type | Comparison | Pseudo-F | R^2^ | p adjusted |
| --- | --- | --- | --- | --- | --- |
| Contramaestre | Feathers | vs Rupert | 18.106 | 0.217 | 0.001 |
|  |  | vs Monmouth | 15.182 | 0.248 | 0.001 |
|  |  | vs Tuckers 1 | 10.552 | 0.143 | 0.001 |
|  |  | vs Tuckers 2 | 6.498 | 0.121 | 0.001 |
|  | Nest soil | vs Rupert | 11.509 | 0.203 | 0.002 |
|  |  | vs Monmouth | 5.966 | 0.238 | 0.002 |
|  |  | vs Tuckers 1 | 5.603 | 0.183 | 0.002 |
|  |  | vs Tuckers 2 | 4.778 | 0.166 | 0.002 |
| Rupert | Feathers | vs Monmouth | 4.514 | 0.091 | 0.001 |
|  |  | vs Tuckers 1 | 4.269 | 0.064 | 0.001 |
|  |  | vs Tuckers 2 | 4.912 | 0.096 | 0.001 |
|  | Nest soil | vs Monmouth | 3.099 | 0.083 | 0.002 |
|  |  | vs Tuckers 1 | 2.111 | 0.050 | 0.007 |
|  |  | vs Tuckers 2 | 2.923 | 0.069 | 0.002 |
| Monmouth | Feathers | vs Tuckers 1 | 3.749 | 0.080 | 0.001 |
|  |  | vs Tuckers 2 | 5.963 | 0.180 | 0.001 |
|  | Nest soil | vs Tuckers 1 | 1.833 | 0.115 | 0.019 |
|  |  | vs Tuckers 2 | 3.168 | 0.195 | 0.002 |
| Tuckers 1 | Feathers | vs Tuckers 2 | 2.199 | 0.047 | 0.003 |
|  | Nest soil | vs Tuckers 2 | 1.981 | 0.094 | 0.015 |

**SUPPLEMENTARY FIGURES**


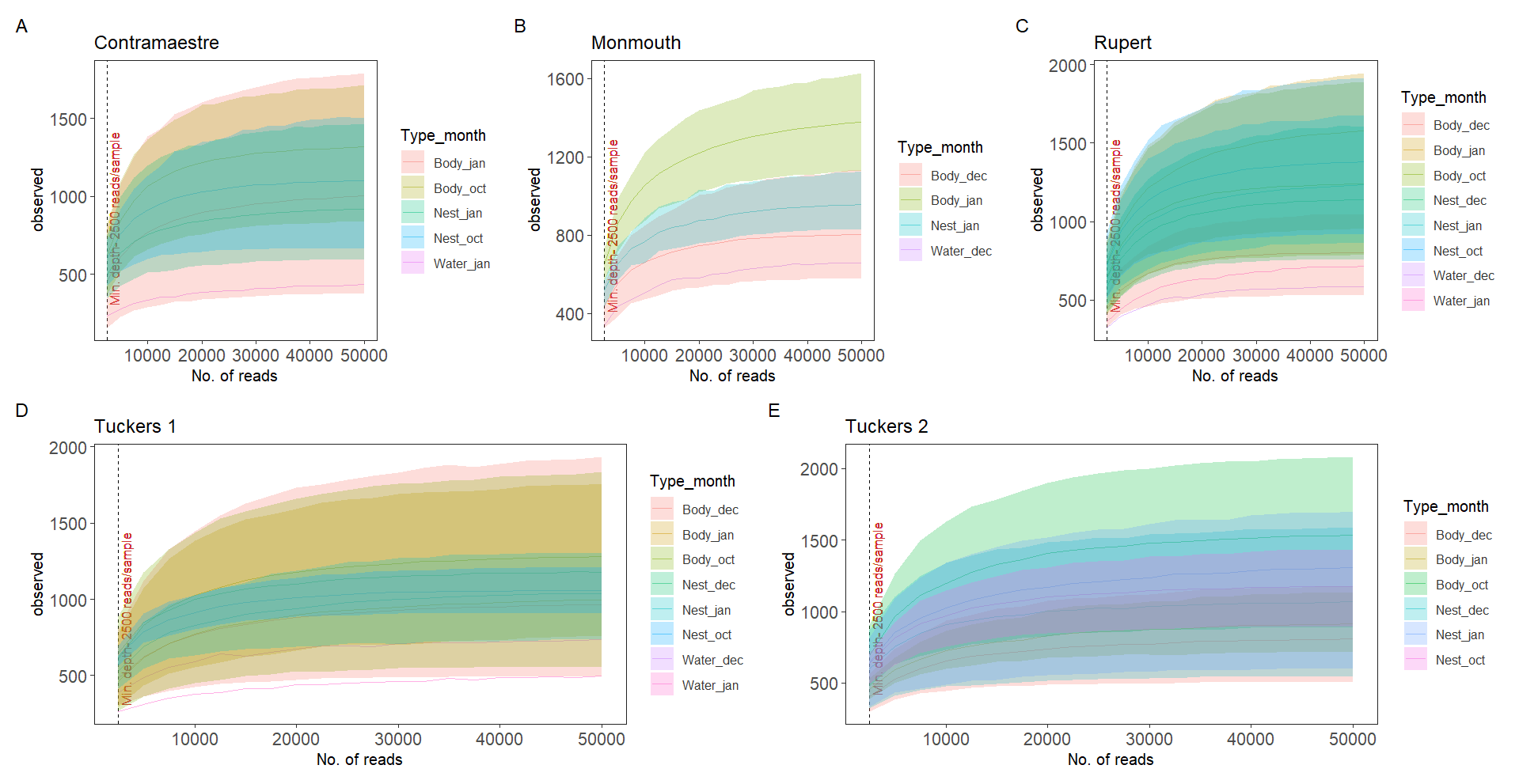


Supplemental Figure 1. Rarefaction plots of each colony: A) Contramaestre, B) Monmouth, C) Rupert, D) Tuckers 1, and E) Tuckers 2.

**
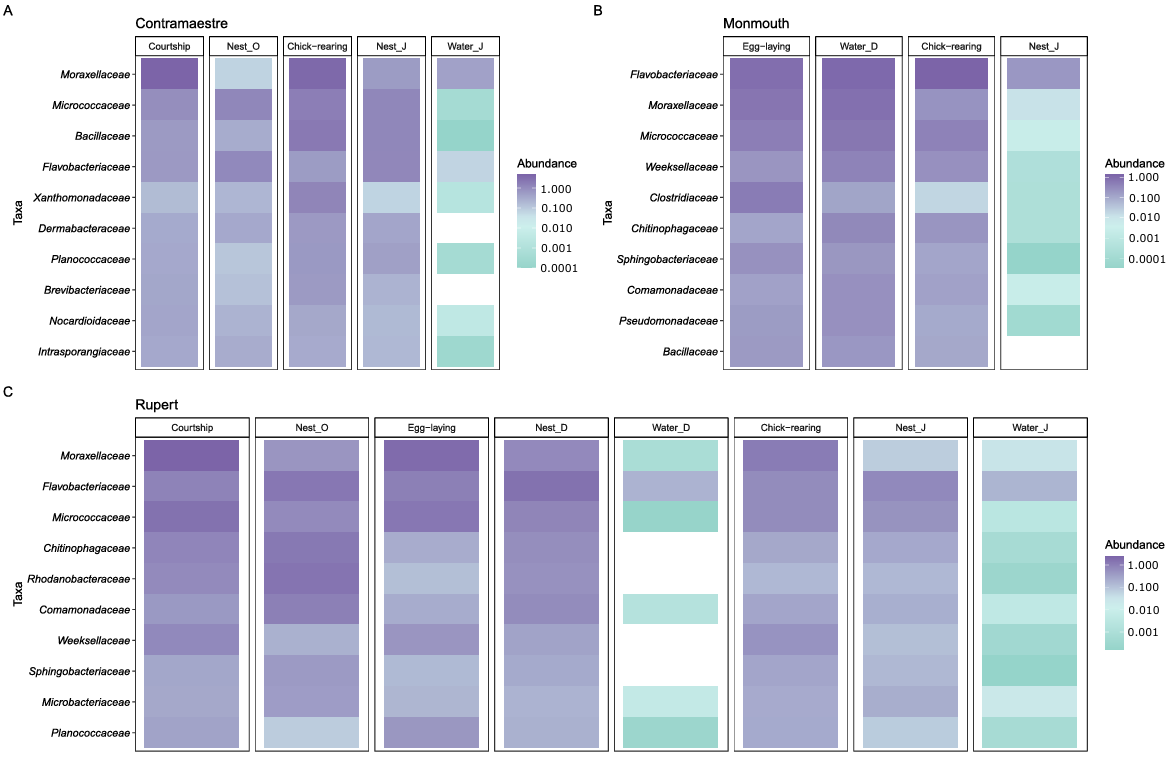
**

Supplemental Figure 2. Relative abundance patterns of the most abundant bacterial families across sample types in A) Contramaestre, B) Monmouth, and C) Rupert. “O” refers to October, “D” refers to December, and “J” refers to January.


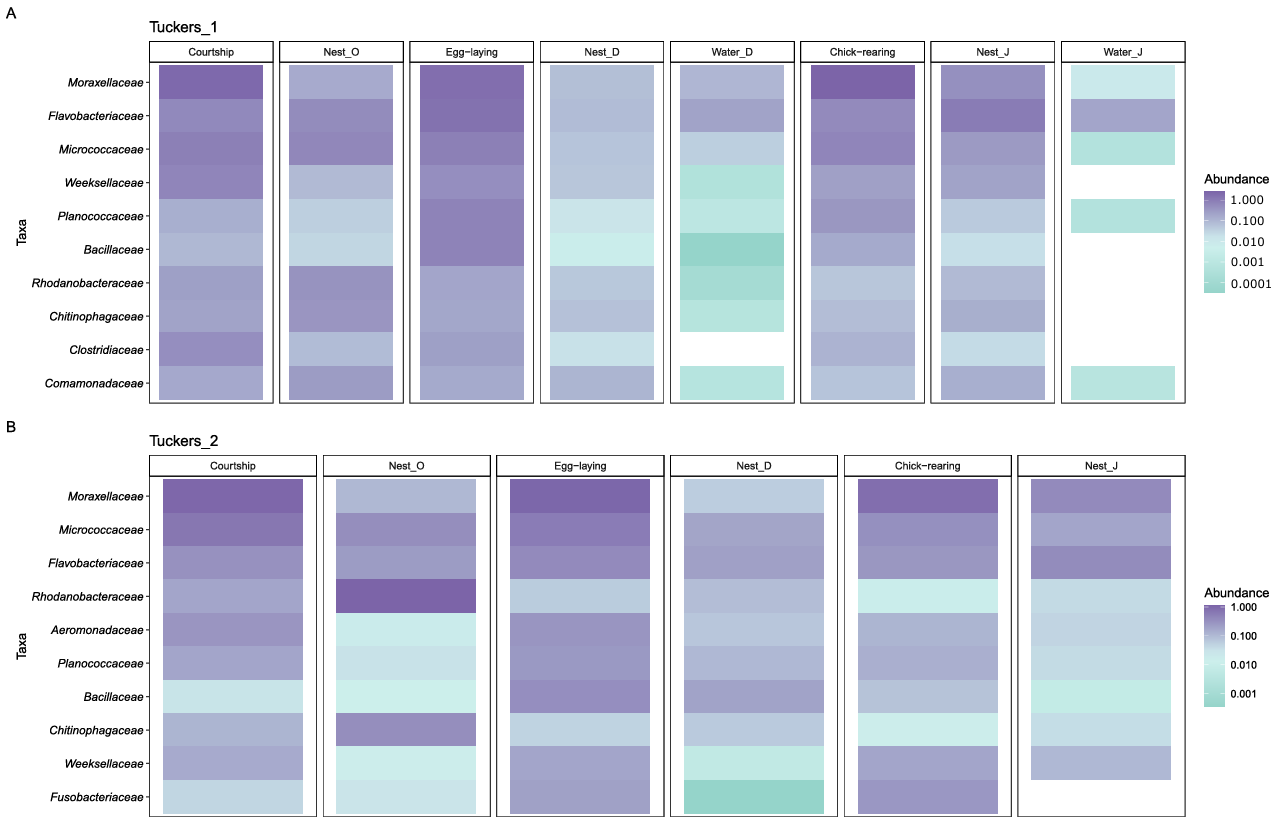
Supplemental Figure 2 (continuation). Relative abundance patterns of the most abundant bacterial families across sample types in A) Tuckers 1 and B) Tuckers 2. “_O” refers to October, “_D” refers to December, and “_J” refers to January.


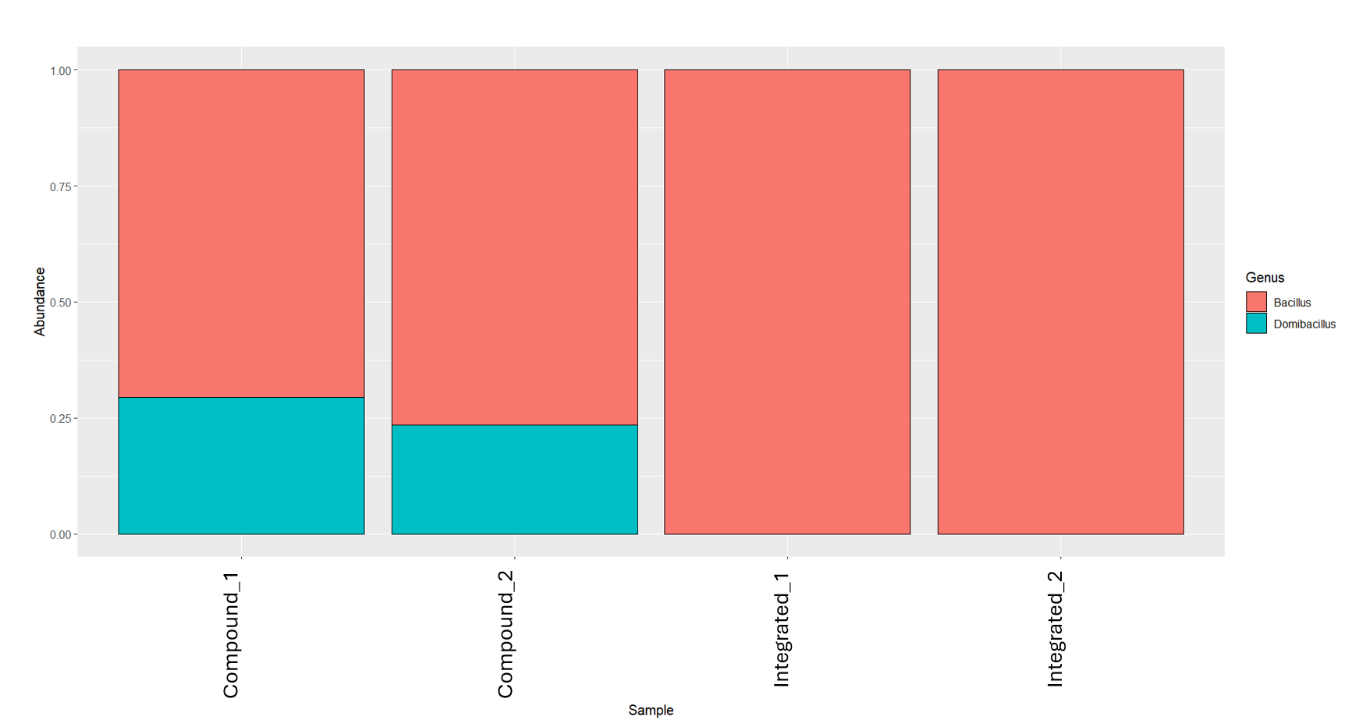


Supplemental Figure 3. Taxonomic composition of Mock communities of different sequencing runs, one with king penguin samples and the other with humpback whale and sealion samples.

**
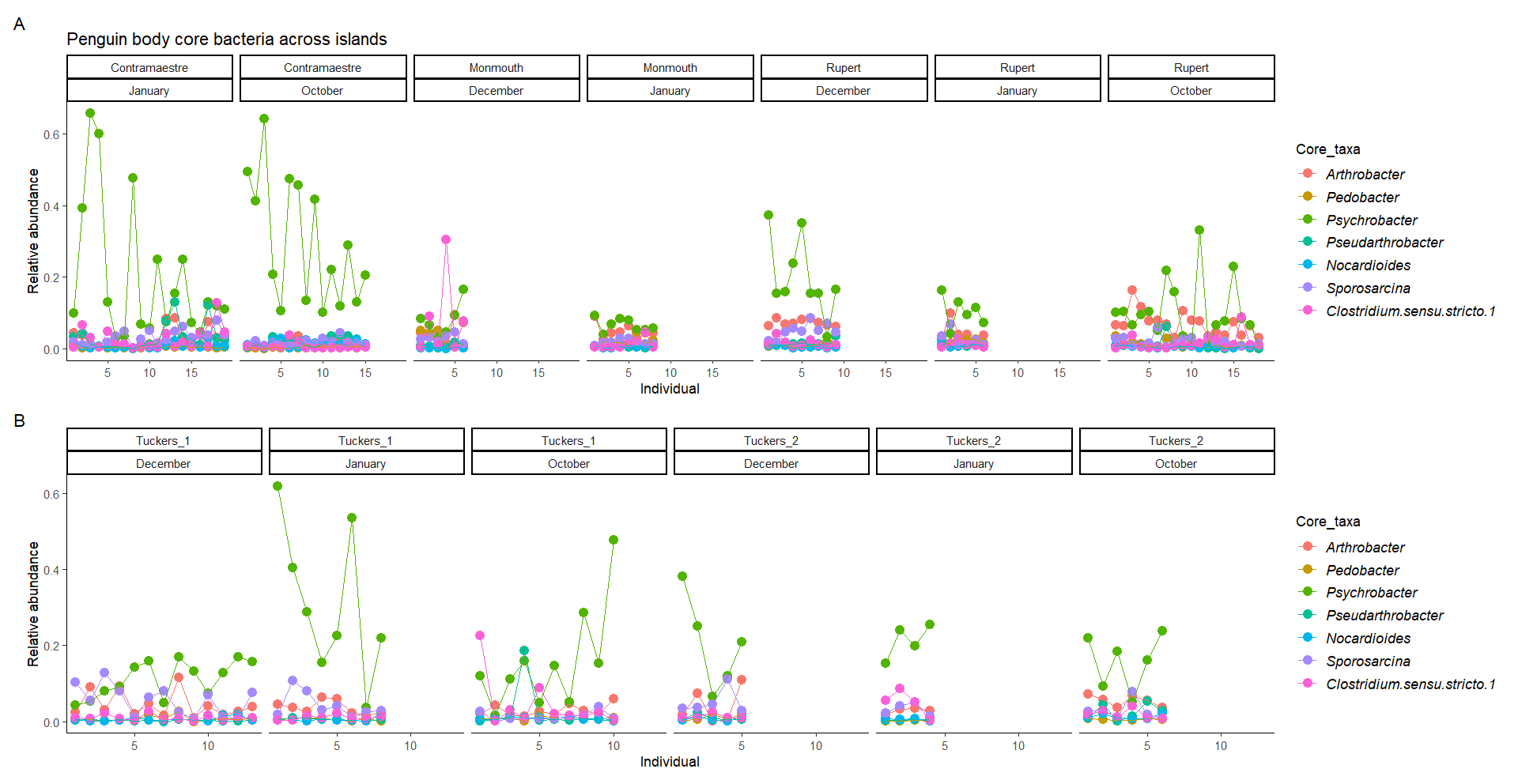
**

Supplementary figure 4. Relative abundance of core bacterial genera with a minimum relative abundance of 0.001, shared at least between 90% of all samples in A) Contramaestre, Monmouth, and Rupert, B) Tuckers 1 and Tuckers 2.


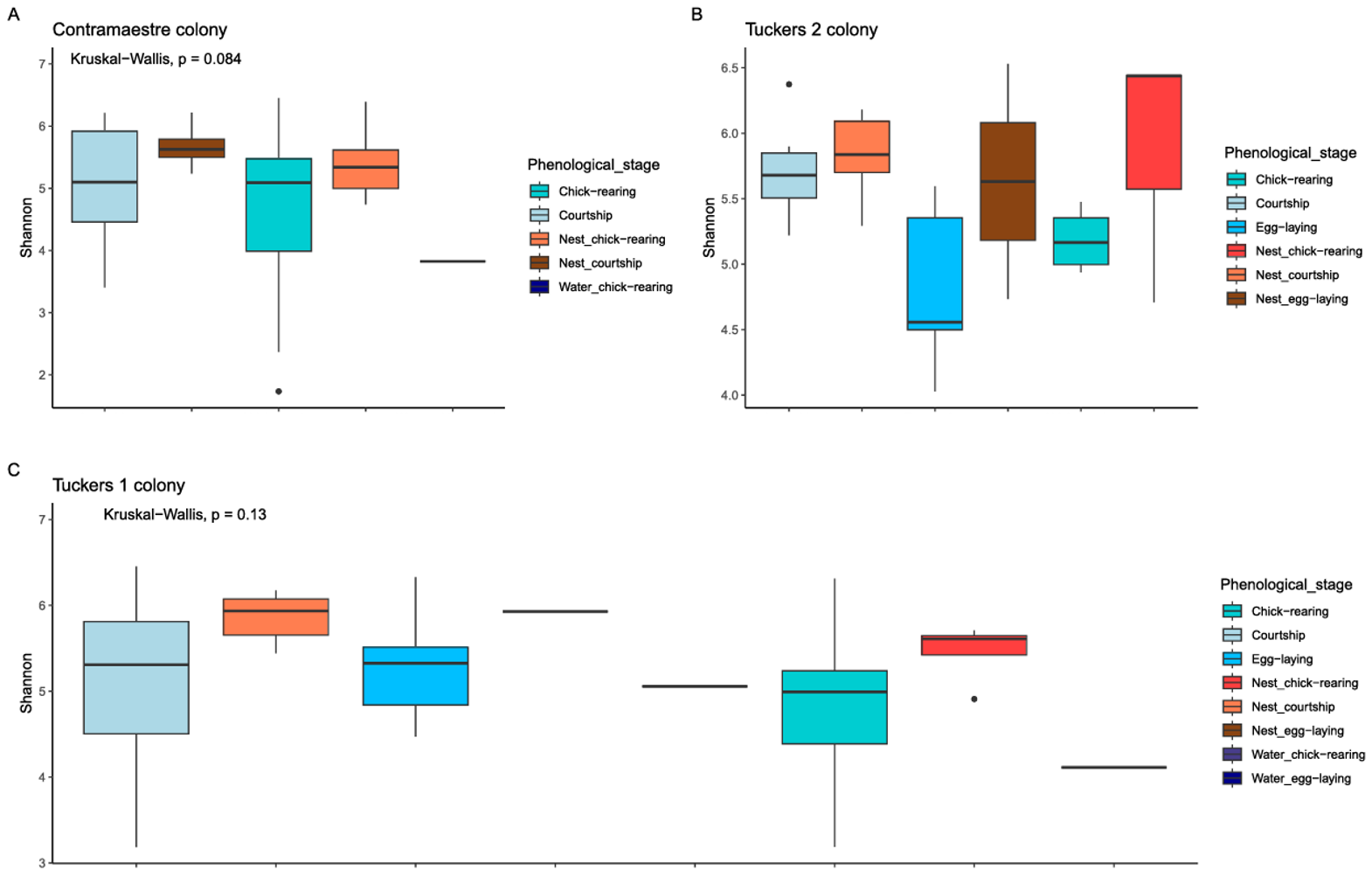


Supplemental figure 5. Microbial alpha diversity patterns in sample types across phenological stages in A) Contramaestre, B), Tuckers 2, and C) Tuckers 1.


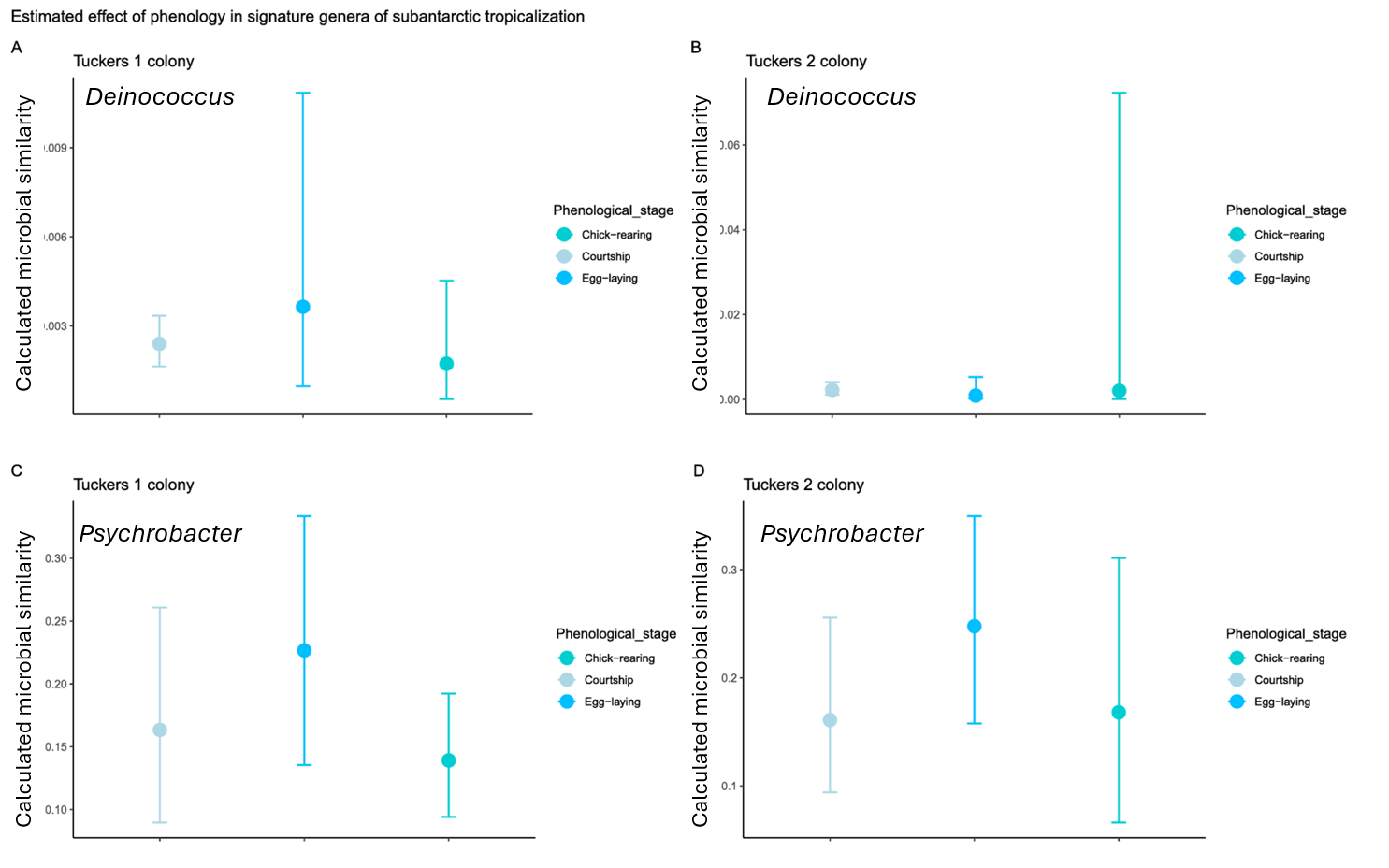


Supplemental figure 6. Nesting colonies where phenology had minimal effects on the relative abundance of selected genera. Phenological dynamics in the relative abundance of *Deinococcous* in the feather microbiota of penguins from A) Tuckers 1 and B) Tuckers 2. Phenological dynamics in the relative abundance of *Psychrobacter* in the feather microbiota of penguins from C) Tuckers 1 and D) Tuckers 2. Dots show estimated average relative abundance and lines represent credibility intervals.


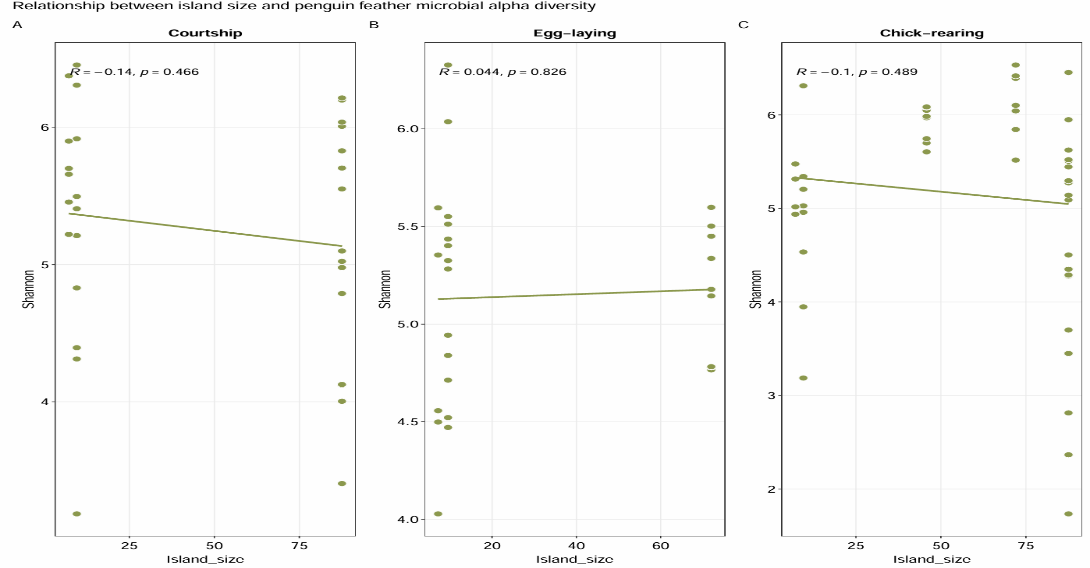


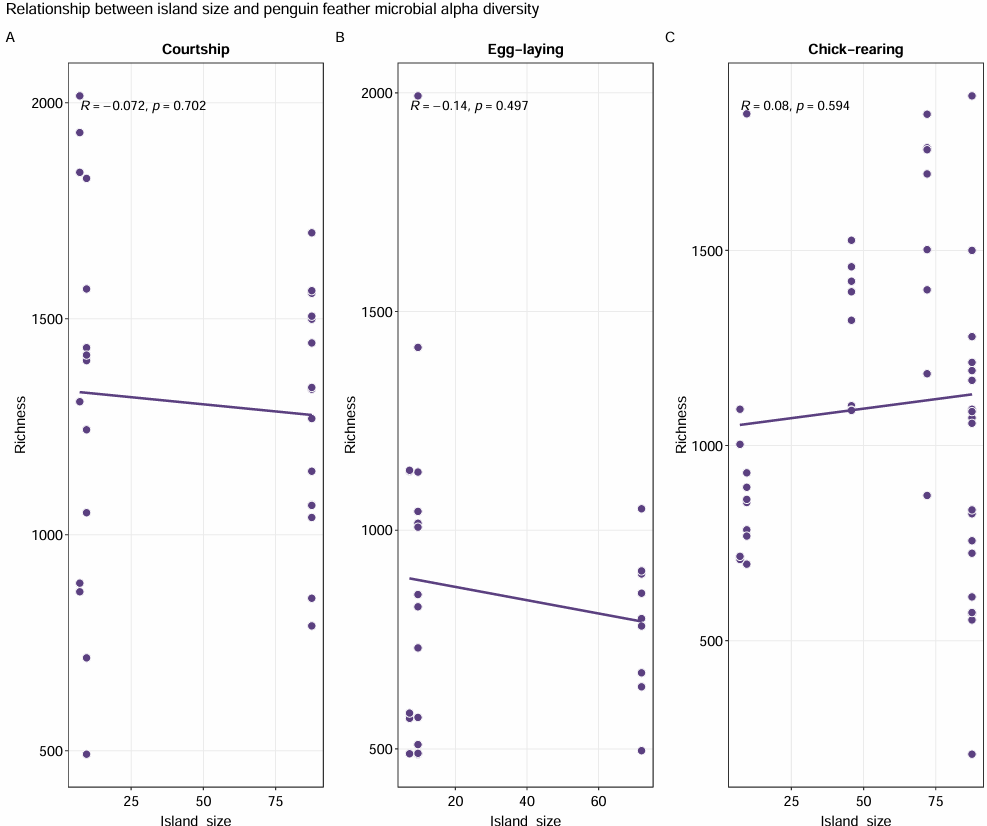


Supplementary Figure 7. Correlation between island size and feather alpha diversity (Shannon, upper panel, and Richness, lower panel) across phenological stages.


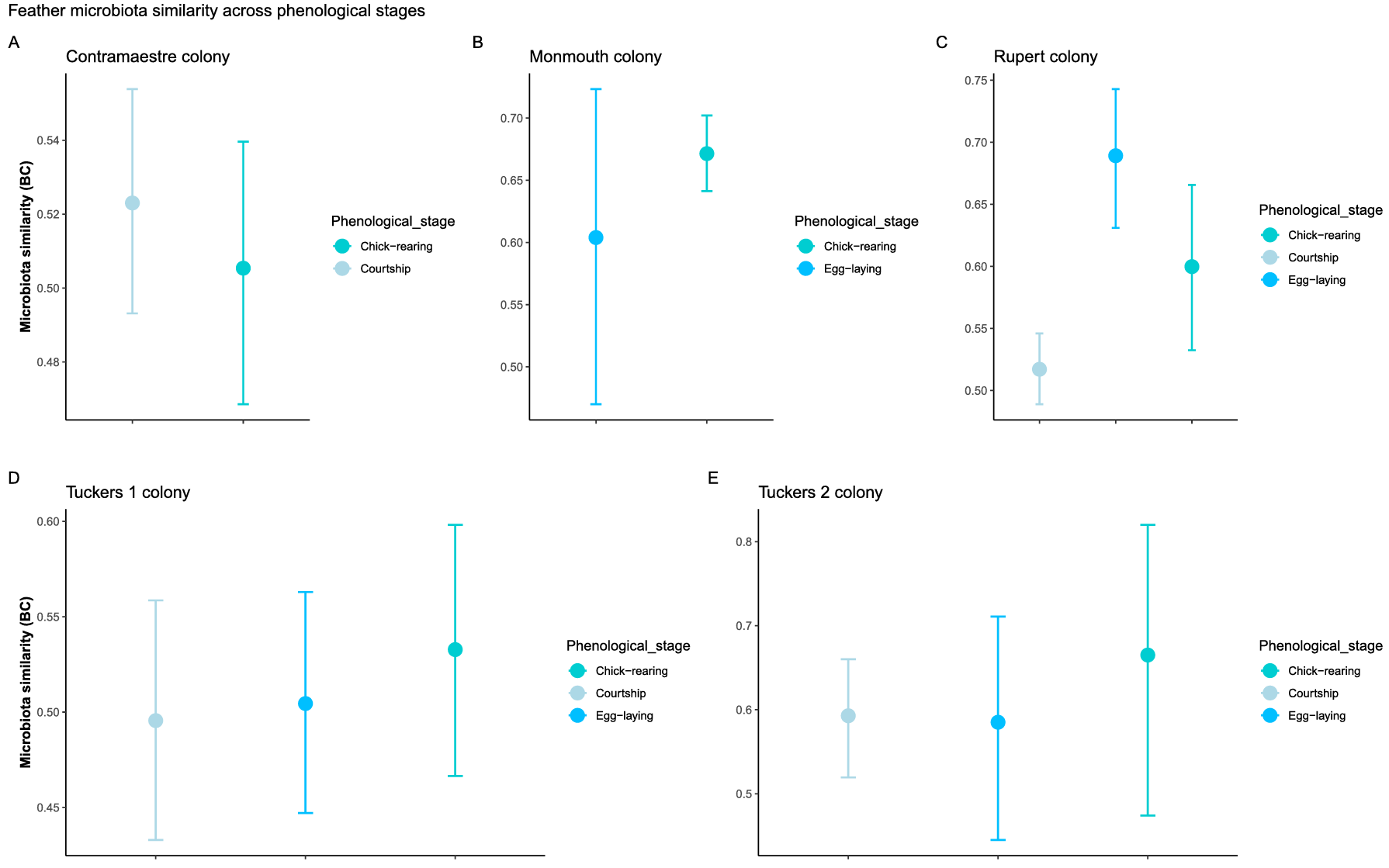


Supplemental figure 8. Feather microbiota compositional similarity (Bray Curtis distance) remains relatively similar in most breeding colonies across phenological stages. Phenological dynamics in the feather microbiota similarity in A) Contramaestre, B) Monmouth, C) Rupert, D) Tuckers 1, and E) Tuckers 2. Dots show estimated average relative abundance and lines represent credibility intervals.


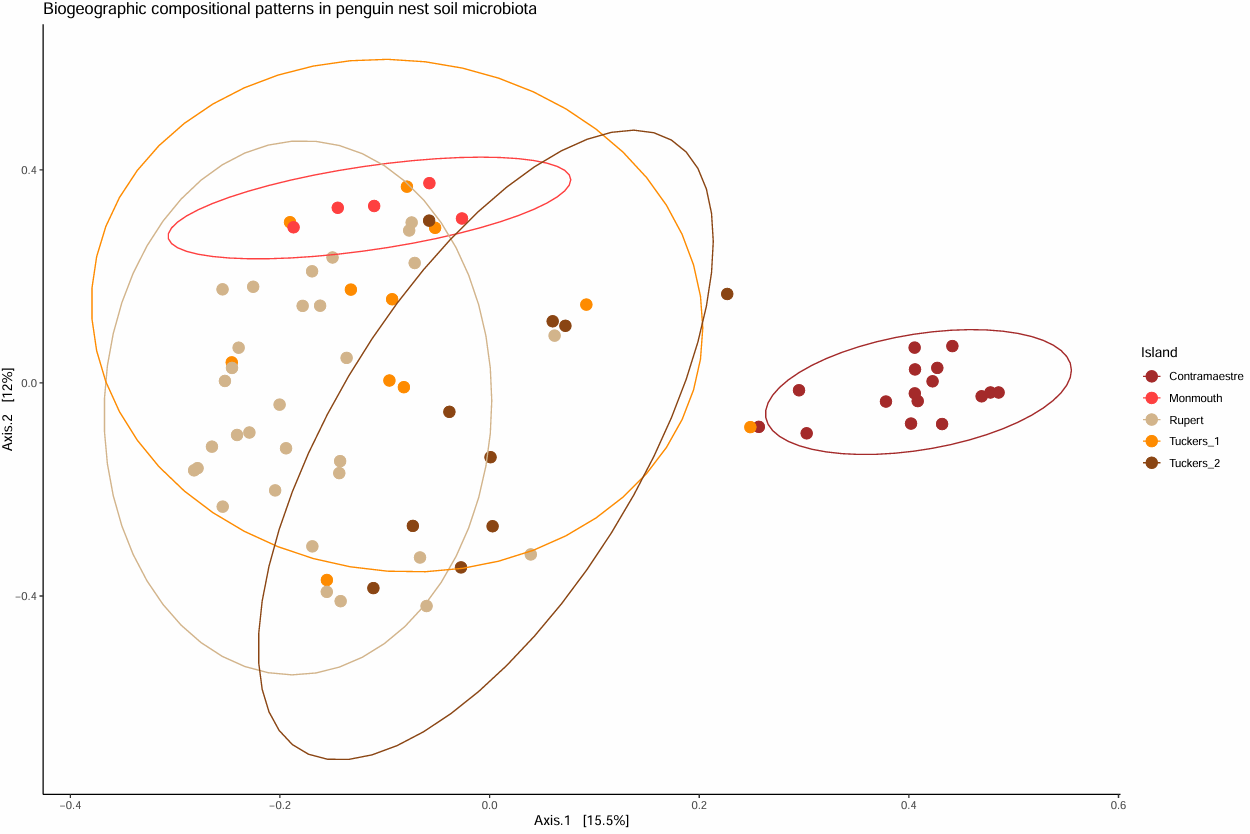


Supplemental figure 9. Geographic ordination in nest soil microbiota of Magellanic penguins.
